# Parp3 assists muscle function and skeletal muscle differentiation by selectively adjusting H3K27me3 enrichment

**DOI:** 10.1016/j.isci.2025.112267

**Published:** 2025-03-25

**Authors:** Zuleyha Yildirim, Aurélia Noll, Kathline Martin-Hernandez, Jean-Christophe Amé, Najat Hanini, Nadia Messaddeq, Isabelle Robert, Bernardo Reina San Martin, Gunn Hildrestrand, Magnar Bjoras, Françoise Dantzer

**Affiliations:** 1 Poly(ADP-ribosyl)ation and Genome Integrity, Laboratoire d’Excellence Medalis, UMR7242, Centre National de la Recherche Scientifique/Université de Strasbourg, Institut de Recherche de l’Ecole de Biotechnologie de Strasbourg, 300 bld. S. Brant, CS10413, 67412 Illkirch, France; 2 IGBMC, Institut de Génétique et de Biologie Moléculaire et Cellulaire, 67400 Illkirch, France; 3 Université de Strasbourg, IGBMC UMR 7104- UMR-S 1258, 67400 Illkirch, France; 4 CNRS, UMR 7104, 67400 Illkirch, France; 5 Inserm, UMR-S 1258, 67400 Illkirch, France; 6 Department of Clinical and Molecular Medicine, Norwegian University of Science and Technology, 7491 Trondheim, Norway; 7 Centre for Embryology and Healthy Development, University of Oslo, 0424 Oslo, Norway

**Keywords:** Molecular mechanism of gene regulation, Epigenetics, Molecular network, Cell biology, Model organism

## Abstract

Poly(ADP-ribose) polymerase 3 (Parp3) is known for its role in DNA repair, mitotic division, and cancer aggressiveness. Still, its physiological roles have yet to be defined. Here, we combined *in vivo* studies using Parp3-deficient mice with *in cellulo* studies to explore the involvement of Parp3 in skeletal muscle function and muscle differentiation. We show that Parp3 contributes to skeletal muscle integrity and promotes myogenic differentiation. Mechanistically, we show that Parp3 promotes the enrichment of the repressive histone mark H3K27me3 onto a panel of selected genes. For some genes, Parp3 also helps the binding of Ezh2, the histone methyltransferase that catalyzes H3K27me3. Moreover, Parp3 ADP-ribosylates Ezh2 *in vitro*. Altogether, these findings unveil Parp3 as a driver of efficient murine skeletal myogenesis *in vitro* and muscle function in young adults, and highlight an epigenetic control of gene expression.

## Highlights

• Parp3 drives efficient skeletal muscle differentiation *in vitro*
• Parp3 contributes to skeletal muscle function and muscle integrity
• Parp3 regulates the abundance of H3K27me3 onto a subset of selected genes

## Graphical abstract

**Figure:**
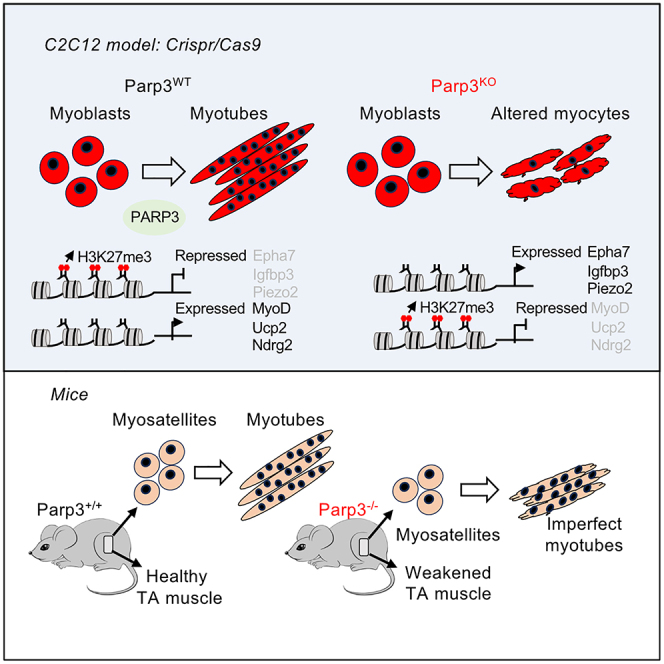

## Introduction

Poly(ADP-ribose) polymerase 3 (Parp3), the third member of the DNA damage-dependent PARP family subgroup, is well recognized for its biochemical and cellular roles in the repair of double-strand breaks by the classical non-homologous end-joining (c-NHEJ) route, in the control of telomere segregation for faithful mitosis and in cancer aggressiveness.^1^^,^^2^^,^^3^^,^^4^ Parp3 catalyzes predominantly mono(ADP-ribosyl)ation, i.e., the addition of single ADP-ribose units onto target proteins, among them Parp3 itself, the repair protein Ku70, or the mitotic components Tankyrase 1 and NuMa.^1^^,^^2^ Furthermore, Parp3 was described to ADP-ribosylate histones at site-specific DNA single-strand breaks or directly 3′ terminal phosphate residues at single and double-strand break termini of DNA molecules, exemplifying its close proximity with chromatin.^5^^,^^6^ Accordingly, there is accumulating evidence that Parp3 also influences transcription by interacting with or modulating the activity of chromatin modifying enzymes with different outcomes on gene expression. In human cancer cells, Parp3 was found to interact with and ADP-ribosylate the histone H3 lysine 9 methyltransferase G9a to restrain the G9a-dependent repression of adhesion and hypoxia-responsive genes.^7^ In the zebrafish, Parp3 was described to have essential functions in ectodermal specification and neural crest development by promoting the transcriptional expression of several developmental genes.^8^ Mechanistically, this role was linked to its association with several polycomb group (PcG) proteins of the PRC2 complex, among others the histone H3 lysine 27 methyltransferase EZH2.^9^

Yet, what is still needed is a greater understanding of the specific physiological functions of Parp3. Originally, Parp3 knockout mice were described viable with no obvious phenotype.^1^ However, further investigations in the mouse brain revealed that Parp3 governs the differentiation of neural stem progenitor cells to astrocytes with implications in the striatum of post-natal mice and shortly after hypoxia-ischemia.^10^ These findings motivated us to interrogate on the potential role of Parp3 in stem cell differentiation in another physiological context.

In the skeletal muscle tissue, differentiation of the resident adult stem cells called satellite cells is vital for efficient skeletal muscle regeneration, a multi-step process switched on in response to various stimuli including exercise, muscle injury, or by degenerative diseases such as muscular dystrophies. The process begins with the proliferation and activation of the satellite cells. The resulting muscle progenitor cells then exit the cell cycle and engage differentiation to multinucleated myofibers, or self-renew.^11^

During the last decade, an important role for epigenetic control of muscle stem cell differentiation has emerged. Increasing studies reveal that the well-known repressive mark H3K27me3 is one of the most frequent histone modification that regulates myogenic differentiation.^12^^,^^13^^,^^14^ Accordingly, additional studies revealed pivotal and coordinated roles of the PRC2 complex required for this modification including the H3 lysine methyltransferase Ezh2 responsible for the catalysis of H3K27me3,^13^ and the PRC1 complex that enforces H3K27me3-mediated repression though in a PRC2-independent manner.^12^ As important for myogenesis, are the antagonistic activities of protein complexes catalyzing H3K27me3 demethylation at muscle specific genes such as the histone demethylase KDM6A (UTX) targeted by the homeobox protein Six4^15^^,^^16^ and regulated by the histone chaperone Spt6.^17^

In the present study, we identified Parp3 as a novel important player in skeletal muscle function and muscle differentiation. Detailed phenotypic investigation in Parp3-deficient mice revealed diminished *Tibialis Anterior* (TA) muscle integrity characterized by reduced muscle strength, the appearance of centrally nucleated myofibers, an increase in the expression of ongoing regeneration markers, and a modified fast-twitch fiber types composition referring to reduced type IIa (Myh2) fibers. Local cold-injury of the TA muscle did not alter the overall capacity of muscle regeneration though the expected induction of early regeneration markers was abrogated. Some of these alterations did not manifest in older animals, likely neutralized by age-related muscle waning. Satellite cells isolated from adult Parp3-deficient mice can differentiate but reproduce reduced expression of *MyoD* and *Myh2* as identified in Parp3^−/−^ TA. *In vitro*, we show that the disruption of Parp3 in C2C12 myoblasts induced an important failure in myogenic differentiation defined by a disorganized actin cytoskeleton, the appearance of degenerative mitochondria and an altered abundance of H3K27me3 on selected genes. Together, these results provide evidence that Parp3 contributes to skeletal muscle function *in vivo*, and fosters efficient skeletal muscle differentiation *in vitro*, mechanistically implying an epigenetic regulation of a subset of genes.

## Results

### The absence of Parp3 in mice weakens TA skeletal muscle function and muscle integrity in young adults in homeostatic conditions

**Figure 1:**
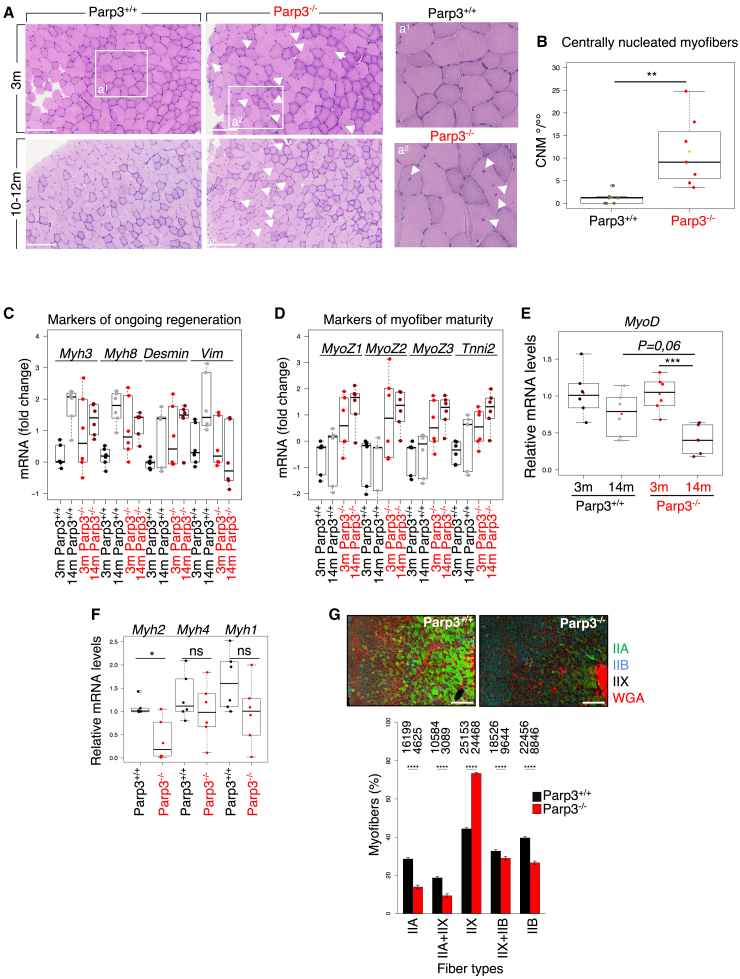
Parp3 contributes to skeletal muscle function and muscle integrity in mice (A) Parp3^−/−^ mice display centrally nucleated myofibers in *Tibialis Anterior* (TA) muscle biopsies. *Left*, representative H&E images of TA muscles from 3 months and 10–12 months male Parp3^+/+^ and Parp3^−/−^ mice. Arrows denote centrally nucleated fibers. Scale bars 125 μm. *Right*, magnified images of boxed areas a1 and a2. (B) Quantification of centrally nucleated myofibers in 3 month-old Parp3^+/+^ and Parp3^−/−^ mice. Data represent the number of CNM/myofibers in *n* = 7 mice/genotype (4 males, 3 females). Statistical differences were calculated using Wilcoxon-Mann-Whitney test ∗∗*p* < 0.01. (C) RT-qPCR expression analysis of *Myh3*, *Myh8*, *Desmin*, and *Vimentin* regeneration-related genes in TA muscles from 3 and 14 months-old Parp3^+/+^ and Parp3^−/−^ mice. *Cmas* mRNA was used for normalization. Data are expressed as fold change (Log 10) with one individual/genotype set to one as a reference and statistical differences were calculated using the Wilcoxon-Mann-Whitney test (*n* = 6 mice per group, 3mParp3^+/+^: 2 males, 4 females; 14mParp3^+/+^: 4 males, 2 females; 3mParp3^−/−^: 3 males, 3 females; 14mParp3^−/−^: 3 males, 3 females). (D) RT-qPCR expression analysis of *Myoz1*, *Myoz2*, *Myoz3*, and *Tnni2* genes associated with myofiber maturity in TA muscles of 3 and 14 months-old Parp3^+/+^ and Parp3^−/−^ mice. *Cmas* mRNA was used for normalization. Data are expressed as fold change (Log 10) with one individual/genotype set to one as a reference and statistical differences were calculated using the Wilcoxon-Mann-Whitney test (*n* = 6 mice per group, 3mParp3^+/+^: 2 males, 4 females; 14mParp3^+/+^: 4 males, 2 females; 3mParp3^−/−^: 3 males, 3 females; 14mParp3^−/−^: 3 males, 3 females). (E) *MyoD* expression is reduced in TA tissues from 14-months-old Parp3^−/−^ mice. RT-qPCR expression analysis of *MyoD* in 3 months and 14 months-old Parp3^+/+^ and Parp3^−/−^ TA biopsies. *Rpl41* mRNA was used for normalization. Data are expressed as relative mRNA levels. Statistical differences were calculated using the Wilcoxon-Mann-Whitney test ∗∗∗*p* < 0.001 (5 < *n* > 7 mice per group, 3mParp3^+/+^: 3 males, 3 females; 14mParp3^+/+^: 3 males, 3 females; 3mParp3^−/−^: 4 males, 3 females; 14mParp3^−/−^: 3 males, 2 females). (F) RT-qPCR expression analysis of *Myh2* (type IIA), *Myh4* (type IIB), and *Myh1* (type IIX) in 3 months-old Parp3^+/+^ and Parp3^−/−^ TA biopsies. *18S* mRNA was used for normalization. Data are expressed as relative mRNA levels. Statistical differences were calculated using the Wilcoxon-Mann-Whitney test ∗*p* < 0,05 (*n* = 6 mice per group, Parp3^+/+^: 4 males, 2 females; Parp3^−/−^: 3 males, 3 females). (G) *Top*, representative immunofluorescence images showing type IIA (green), IIB (blue), IIX (unlabeled, dark), and WGA (red) fibers in TA muscles from 3 months-old Parp3^+/+^ and Parp3^−/−^ mice. Scale bars 200 μm. *Bottom*, quantification of fiber type distribution in TA muscles from 3 months-old Parp3^+/+^ and Parp3^−/−^ mice. The total number of fiber types counted in each category is indicated. (*n* = 4 mice/genotype, 2 males, 2 females). Statistical differences were calculated using the Wilcoxon-Mann-Whitney test ∗∗∗∗*p* < 0.0001. See also Figure S1.

We have previously shown that Parp3 promotes astroglial differentiation and regeneration of the striatum upon cerebral hypoxia-ischemia.^10^ We were interested to determine whether Parp3 serves in another physiological system involving stem cell differentiation. We aimed to investigate muscle integrity in young and old adult Parp3^−/−^ mice compared to Parp3^+/+^ counterparts. H&E staining of TA muscles from naive young adults (3 months old) revealed the appearance of centrally nucleated myofibers in Parp3^−/−^ mice but with no other signs of degeneration, no change in muscle fiber diameters, and no obvious aggravation in old (>10 months) mice (Figures 1A and 1B; Figures S1A and S1B). Accordingly, grip strength was reduced in 3 months-old Parp3^−/−^ mice and was persistently lower than that of Parp3^+/+^ mice while not worsening with age (Figure S1C). Centrally nucleated fibers are generally recognized as regenerated myofibers. They are meant to compensate the muscle impairment induced by muscle damage and muscle dystrophies. Thus for a deeper assessment of myofiber regeneration and muscle integrity, we carefully analyzed expression patterns of several muscle regeneration-related genes in both young and old Parp3^+/+^ versus Parp3^−/−^ mice.^18^ Transcript expression of embryonic or perinatal myogenic markers (*Myh3*, *Myh8*, *Desmin*, and *Vimentin*) characterizing ongoing regeneration, showed an enhanced expression of these markers in old compared to young Parp3^+/+^ mice, an observation correlated with skeletal muscle decline during aging (Figure 1C). Interestingly, this age-related increase appeared less pronounced in the Parp3^−/−^ mice. For *Myh3*, *Myh8*, and *Desmin*, this result can be explained by a perceptible increased expression of these genes in already young Parp3^−/−^ mice compared to age-matched Parp3^+/+^ mice suggesting continuous regeneration in the young Parp3^−/−^ TA muscles (Figure 1C). Transcript expression of terminal differentiation markers reflecting myofiber maturity (*Myoz1*, *Myoz2*, *Myoz3*, and *Tnni2*) also revealed a tendency toward higher expression in Parp3^−/−^ mice versus Parp3^+/+^ mice that reflects a substantial extend of muscle regeneration (Figure 1D). Together, these results suggest sustained regeneration starting in the young Parp3^−/−^ TA muscles and maintained during aging, corroborating the presence of centrally nucleated myofibers (Figures 1A and 1B) and muscle weakness (Figure S1C). Since the myogenic regulatory factor MyoD has been shown to play a unique role in muscle regeneration, we then analyzed the expression levels of *MyoD* in both young and aged Parp3^+/+^ versus Parp3^−/−^ mice. While no difference was detected in the young mice, a noticeable decrease in *MyoD* levels was detected in the old Parp3^−/−^ mice relative to the aged-matched Parp3^+/+^ mice (Figure 1E).

Among the major indicators of muscle function and muscle integrity, the amount and type of MyHC fibers are also of key relevance. The slow-twitch type I fibers (MyHC-I) are not detected in TA muscles. Thus, to investigate if young Parp3-deficient mice show alterations in fiber type composition, we analyzed the expression levels of the major fast-twitch (type II) fibers *Myh2* (MyHC-IIA), *Myh4* (MyHC-IIB), and *Myh1*(MyHC-IIX). We detected a significant decrease in the expression level of *Myh2* (MyHC-IIA) only (Figure 1F). To verify this result further, we performed immunofluorescence for MyHC-IIA, MyHC-IIB, and MyHC-IIX isoforms in sections of Parp3^+/+^ and Parp3^−/−^ TA muscles. We validated a significant decrease in the number of type IIA fibers, we also detected a significant decrease in the type IIB fibers and in contrast we measured a significant increase in the type IIX fibers suggesting a progressive compensation (Figure 1G). Overall, these findings revealed important changes in type II fiber type composition in the Parp3^−/−^ TA muscles that can contribute to their fragility.

### Parp3-deficient satellite cells can differentiate but myotubes display reduced expression of *MyoD* and *Myh2*

**Figure 2:**
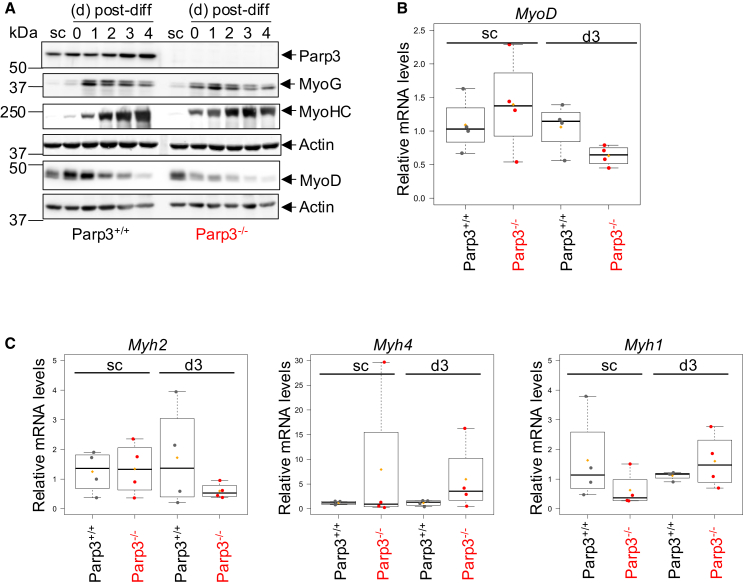
Parp3-deficient muscle satellite cells efficiently differentiate to myotubes but recapitulate diminished expression of MyoD and *Myh2* as in Parp3^−/−^ TA (A) Western blot analysis of Parp3, the myogenic differentiation markers MyoG and MyoHC, the master transcription factor MyoD and actin as loading control in satellite cells grown as myoblasts (sc, subconfluent) or induced to differentiate for 4 days. Myosatellite cells were isolated from Parp3^+/+^ and Parp3^−/−^ 3-months-old female mices. A representative experiment out of 5 is shown. See also Figure S7. (B) RT-qPCR expression analysis of *MyoD* in the Parp3^+/+^ versus the Parp3^−/−^ subconfluent (sc) satellite cells and 3 days post-differentiation. *Cmas* mRNA was used to normalize loading variability. Data are expressed as relative mRNA levels and as mean of two independent differentiation experiments performed each with 4 different satellite cells isolated from 4 mice/genotype (2 males, 2 females). (C) RT-qPCR expression analysis of *Myh2* (type IIA), *Myh4* (type IIB), and *Myh1* (type IIX) in the Parp3^+/+^ versus the Parp3^−/−^ subconfluent (sc) satellite cells and 3 days post-differentiation. *Cmas* mRNA was used to normalize loading variability. Data are expressed as relative mRNA levels and as mean of two independent differentiation experiments performed each with 4 different satellite cells isolated from 4 mice/genotype (2 males, 2 females). Statistical differences were analyzed using the Wilcoxon-Mann-Whitney test.

Since correct muscle regeneration depends on the ability of satellite cells to differentiate to myotubes, we decided to explore the myogenic differentiation potential of myosatellite cells isolated from Parp3^+/+^ and Parp3^−/−^ TA muscles (Figure 2).

We first monitored the expression profile of Parp3. Parp3 increased gradually throughout differentiation potentially indicating a contribution of Parp3 in myogenic differentiation (Figures 2A and S7). To explore this hypothesis, we analyzed the effect of Parp3 loss on the expression levels of the two established differentiation markers MyoG and MyoHC and the myogenic regulatory factor MyoD. Relative to Parp3^+/+^ controls, Parp3^−/−^ satellite cells showed similar expression profiles of MyoG and MyoHC throughout myogenic differentiation, but the expression profile of MyoD was markedly reduced (Figure 2A). Consistently, and in support of the reduced *MyoD* detected in Parp3^−/−^ TA, Parp3^−/−^ differentiated satellite cells displayed diminished transcriptional expression of *MyoD* compared to Parp3^+/+^ controls (Figure 2B).

Moreover, to substantiate on the alterations in fiber type composition identified in Parp3-deficient TA, we verified the expression levels of *Myh2*, *Myh1*, and *Myh4*. Differentiated Parp3^−/−^ satellite cells mirrored the reduced expression of *Myh2*, while *Myh1* and *Myh4* were not altered (Figure 2C).

Collectively, these data indicate that Parp3^−/−^ satellite cells present a normal capacity to differentiate to myotubes but recapitulate the alterations in *MyoD* and *Myh2* expression identified in Parp3^−/−^ TA.

### The absence of Parp3 perturbs the expression profile of ongoing regeneration markers during skeletal muscle regeneration induced by cold-injury

**Figure 3:**
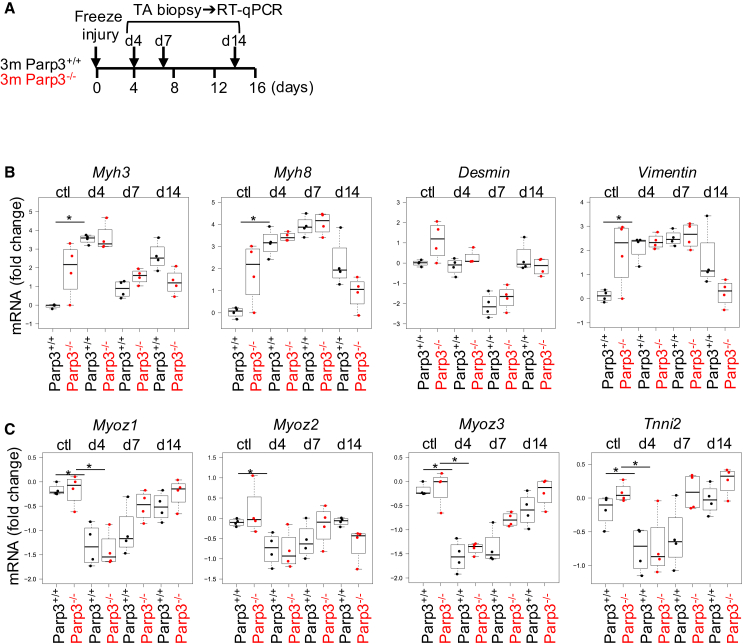
The absence of Parp3 modifies the dynamic of expression of ongoing regeneration-related genes upon acute muscle injury in young adults (A) Diagram of the experimental approach. Parp3^+/+^ and Parp3^−/−^ mice were exposed to freeze-induced muscle injury. TA muscle biopsies were taken from non-injured control mice (ctl) and at d4, d7, d14 post-injury and processed for RT-qPCR. (B) RT-qPCR expression analysis of *Myh3*, *Myh8*, *Desmin*, and *Vimentin* regeneration-related genes in TA muscles from 3 months-old Parp3^+/+^ and Parp3^−/−^ mice left non-injured (ctl) and at the indicated time points throughout muscle regeneration. (C) RT-qPCR expression analysis of *Myoz1*, *Myoz1*, *Myoz3*, and *Tnni2* genes associated with myofiber maturity in TA muscles from 3 months-old Parp3^+/+^ and Parp3^−/−^ mice left non-injured (ctl) and at the indicated time points throughout muscle regeneration. *Cmas* mRNA was used for normalization. Data are expressed as fold change (Log 10) with one individual/genotype set to one as a reference and were analyzed using Wilcoxon-Mann-Whitney test ∗*p* < 0,05. (*n* = 4 mice/condition, Parp3^+/+^ctl: 4 females, Parp3^−/−^ctl: 4 females, Parp3^+/+^d4: 4 males, Parp3^−/−^d4: 2 males, 2 females, Parp3^+/+^d7: 1 male, 3 females, Parp3^−/−^ d7: 4 females, Parp3^+/+^d14: 1 male, 3 females, Parp3^−/−^d14: 3 males, 1 female). See also Figure S2.

Having shown that Parp3-deficient mice display continuous regeneration in homeostatic conditions, we examined the efficiency of muscle regeneration upon acute injury. Local TA muscle damage was induced by cryoinjury^19^ (Figure 3A). H&E staining did not reveal obvious signs of overall defective skeletal muscle regeneration in young Parp3^−/−^ versus Parp3^+/+^ mice at d4, d7, d14 post-injury (Figure S1D). Thus, for a deeper and quantitative assessment of myofiber regeneration, we carefully analyzed the expression patterns of the early and terminal muscle regeneration-related genes as aforementioned (Figures 1C and 1D). Giving support to Figure 1C, intact Parp3^−/−^ TA displayed a noticeable increase in the expression levels of the ongoing regeneration markers *Myh3*, *Myh8*, *Desmin*, and *Vimentin* compared to Parp3^+/+^ TA, explained by constant regeneration in the Parp3^−/−^ muscles. Consequently, while the expression levels of these markers were highly induced upon muscle injury in young Parp3^+/+^ TA and then gradually declined thereafter when muscle repair proceeds as previously published,^18^ this dynamic was lost in the young Parp3^−/−^ TA (Figure 3B). In contrast, transcript expression of terminal differentiation markers characterizing myofiber maturity (*Myoz1*, *Myoz2*, *Myoz3*, and *Tnni2*) revealed a comparable profile in both Parp3^+/+^ and Parp3^−/−^ TA with a significant downregulation at day 4 upon muscle injury compared to untreated mice, followed by a gradual upregulation as regeneration proceeds (Figure 3C). The analysis of these genes in regeneration experiments performed on old animals showed no difference in the transcript expression profiles of both sets of genes likely explained by a progressive erasure of the phenotype with aging (Figure S2).

Together, these results indicate that the absence of Parp3 slightly perturbs injury-induced muscle regeneration by altering the expression profile of the ongoing regeneration markers.

### Parp3 promotes skeletal myogenesis *in vitro*

**Figure 4:**
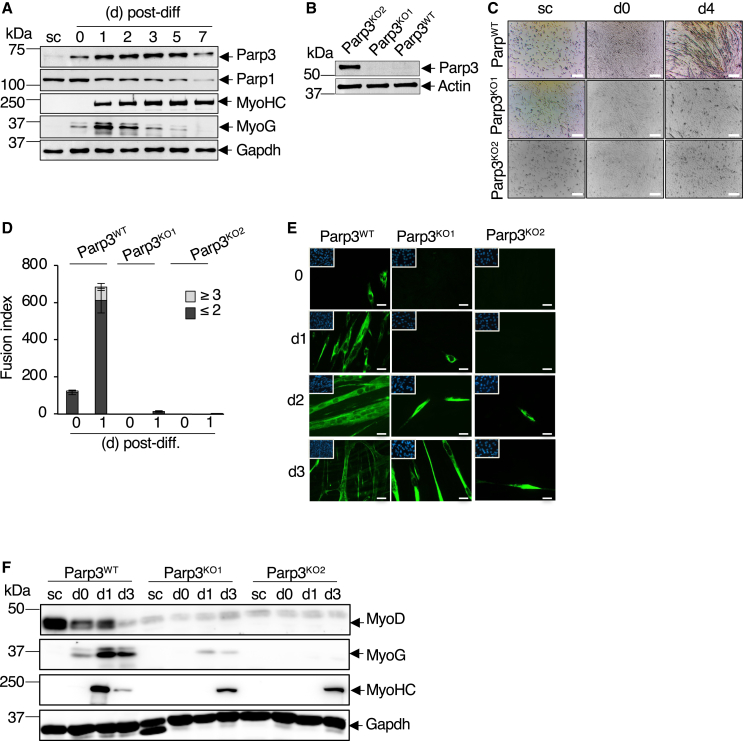
Parp3 is required for efficient differentiation of C2C12 myoblasts to myotubes (A) Nuclear protein extracts from proliferating myoblasts (sc, subconfluent), confluent myoblasts (day 0) and at the indicated time points throughout differentiation (day 1–7) were analyzed for the expression levels of Parp3, Parp1 and the myogenic markers of differentiation MyoG and MyoHC. Gapdh was used as standard control. The figure shown is a representative experiment of three replicates. See also Figure S8. (B) Western blot analysis of Parp3 expression in the wild-type (Parp3^WT^) and the two Parp3^KO1^ and Parp3^KO2^ clones engineered by the CRISPR-Cas9 approach and selected upon screening and sequence analysis. Actin was used as standard control. See also Figure S9. (C) The Parp3^WT^ and the two Parp3-deficient Parp3^KO1^ and Parp3^KO2^ C2C12 myoblasts were induced to differentiate into myotubes for 4 days. Representative bright field images of the proliferating myoblasts (sc, subconfluent), the confluent myoblasts (day 0) and the differentiated myotubes (day 4) are shown. Scale bars: 100 μm. (D) Quantification of the fusion index of the Parp3^WT^, Parp3^KO1^, and Parp3^KO2^ cells at day 0 and day 1 of differentiation. Fusion index represents the mean number (+/− S.D.) of MyoHC-positive cells with two or less nuclei compared to 3 and more nuclei from three independent experiments. Statistical differences were analyzed using Student’s t test. ≤ 2: Parp3^WT^ versus Parp3^KO1^(d0) ∗∗*p* = 0.008; Parp3^WT^ versus Parp3^KO2^ (d0) ∗∗*p* = 0.008; Parp3^WT^ versus Parp3^KO1^(d1) ∗∗*p* = 0,00669; Parp3^WT^ versus Parp3^KO2^ (d1) ∗∗*p* = 0,00662; ≥3: Parp3^WT^ versus Parp3^KO1^(d0) ∗*p* = 0,0438; Parp3^WT^ versus Parp3^KO2^ (d0) ∗*p* = 0,0438; Parp3^WT^ versus Parp3^KO1^(d1) ∗*p* = 0,0416; Parp3^WT^ versus Parp3^KO2^ (d1) ∗*p* = 0,0416. (E) Representative photographs of the Parp3^WT^, Parp3^KO1^, and Parp3^KO2^ cells stained with a green-labeled anti-MyoHC antibody at the indicated time points throughout differentiation (used in D). Inserts show the representative fields of Dapi-stained nuclei. Scale bars: 20 μm. (F) Comparative western blot analysis of the myogenic differentiation markers MyoG and MyoHC and the master transcription factor MyoD in the Parp3^WT^ versus the Parp3^KO1^ and Parp3^KO2^ proliferating myoblasts (sc, subconfluent) and at the indicated time points throughout differentiation. Gapdh was used as a standard control. The figure shown is a representative experiment of three replicates. See also Figures S3 and S10.

**Figure 5:**
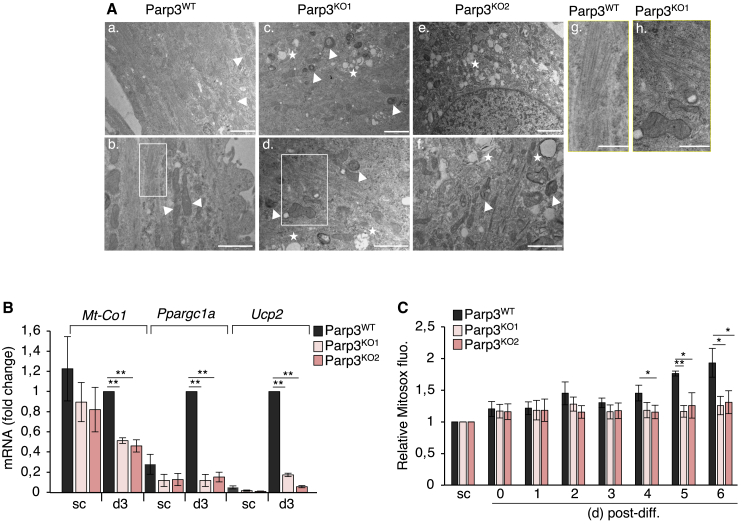
The absence of Parp3 induces cytoskeleton disorganization, mitochondrial dysfunction and the accumulation of autophagic-like vacuoles at day 3 post-differentiation (A) Representative transmission-electron microscopy imaging showing the ultrastructure of the Parp3^WT^ (a and b), Parp3^KO1^ (c and d), and Parp3^KO2^ (e and f) cells at day 3 post-differentiation. Two magnifications are shown. Scale bars, 2 μm (a, c, and e); Scale bars, 1 μm (b, d, and f). White arrows show healthy mitochondria in Parp3^WT^ myotubes and degenerating mitochondria in Parp3^KO^ cells. Stars show autophagic vacuoles. In g,h magnified images of boxed areas focusing on the mis-organized actin fibers in Parp3^KO1^ (h) while fibers are organized as parallel sheets in Parp3^WT^ (g). (B) RT-qPCR analysis of *Mt-Co1*, *Ppargc1a* and *Ucp2* mitochondrial genes in the Parp3^WT^ versus the Parp3^KO1^ and Parp3^KO2^ subconfluent (sc) cells and post-differentiation (d3). *β*2 *microglobuline* mRNA was used to normalize loading variability. Data are expressed as fold change and as mean (+/− S.D.) of three independent experiments with the Parp3^WT^ d3 set to 1. Statistical differences were analyzed using Student’s t test ∗∗*p* < 0,01. (C) Measurement of mitochondrial ROS production in the Parp3^WT^ versus the Parp3^KO1^ and Parp3^KO2^ subconfluent (sc) cells and at the indicated time points throughout myogenic differentiation. Bar graph depicts the fold change relative to subconfluent cells (sc) set to 1. Values represent means (+/− S.D.) of three independent experiments. Statistical differences were analyzed using Student’s t test ∗*p* < 0.05; ∗∗*p* < 0.01. See also Figure S4.

To gain a deeper understanding of the function of Parp3 in skeletal myogenesis, we capitalized on mouse C2C12 myoblasts, a well-characterized *in vitro* muscle differentiation model. We first analyzed the expression profile of Parp3 during myogenesis. In echo to satellite cells (Figure 2A), the protein expression level of Parp3 was moderate in the proliferating myoblasts, but significantly increased throughout their differentiation to myotubes (Figure 4A). In contrast, the expression of Parp1 was high in subconfluent myoblasts and gradually declined throughout myogenic differentiation (Figures 4A and S8). To evaluate whether Parp3 plays a role in regulating *in vitro* myogenesis, we then inactivated Parp3 in the C2C12 myoblasts using the CRISPR-Cas9 strategy (Figure S3). Two independent Parp3^KO^ clones were isolated (Figures 4B and S9). In both clones, knockout of Parp3 significantly impaired the capacity of C2C12 myoblasts to form myotubes (Figure 4C). The fusion index was markedly reduced. No multinucleated myotubes were obtained and only a few binucleated myocytes were detected (Figures 4D and 4E). The protein expression levels of myosin heavy chain (MyoHC), the myogenic regulatory factor Myogenin (MyoG), and the myogenic transcription factor MyoD were drastically reduced (Figures 4F and S10). Ultrastructural analysis of Parp3^KO^ immature myocytes revealed profound alterations of the actin cytoskeleton architecture characterized by short and disorganized fibers while in Parp3^WT^ myotubes, the actin cytoskeleton was arranged in long bundles crossing the cell body (Figure 5A). Abnormal myocytes also displayed degenerative mitochondria and the accumulation of autophagic-like vacuoles (Figure 5A). Altered mitochondrial function was confirmed by a significant decrease in the mRNA expression levels of the mitochondrial markers *Mt-Co1*, *Ppargc1a*, and *Ucp2* (Figure 5B), a reduced capacity to produce mitochondrial reactive oxygen species (ROS) throughout differentiation (Figure 5C), and transiently a lower expression of the mitochondrial respiratory complex II and V suggesting weakened oxidative phosphorylation (Figure S4A). Autophagy was verified by the augmented conversion of LC3-I to LC3-II throughout the differentiation of Parp3^KO1^ myoblasts compared to Parp3^WT^ (Figure S4B). Dosage of the mtDNA revealed a notable increase in the mtDNA copy number in the Parp3^KO^ myocytes interpreted as a compensatory response to attenuate the effect of mitochondrial dysfunction (Figure S4C).

### Restoration of Parp3’s catalytic activity in myoblasts rescues skeletal myogenesis

**Figure 6:**
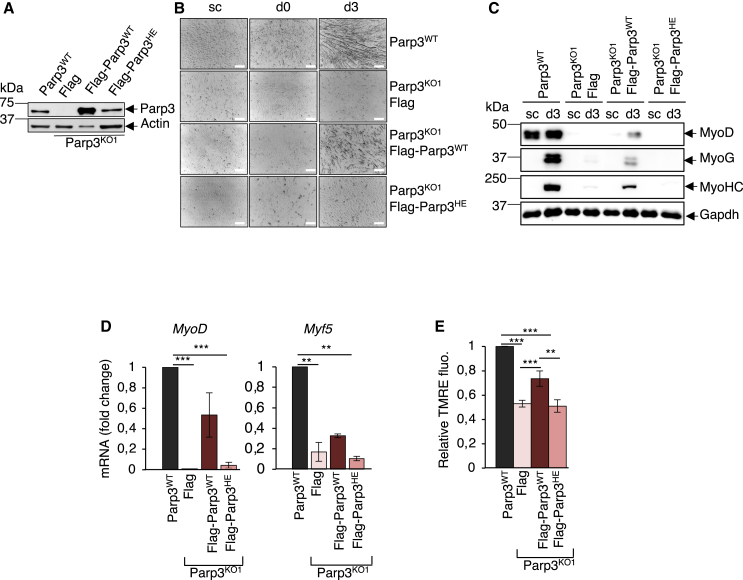
Impaired differentiation of the Parp3-knockout C2C12 cells is rescued by the re-expression of the catalytically active Parp3 but not by the re-expression of a catalytically dead Parp3 (A) Western blot analysis of Parp3 expression in the Parp3^WT^ and Parp3^KO1^ C2C12 cells with a stable expression of either the FLAG control (FLAG), FLAG-Parp3^WT^, or FLAG-Parp3^HE^. Actin is used as loading control. See also Figure S11. (B) The Parp3^WT^, Parp3^KO1^-FLAG, Parp3^KO1^-FLAG-Parp3^WT^, and Parp3^KO1^-FLAG-Parp3^HE^ C2C12 myoblast cell lines were induced to differentiate into myotubes for 3 days. Representative bright field images of the proliferating myoblasts (sc, subconfluent), the confluent myoblasts (day 0) and the differentiated myotubes (day 3) are shown. Scale bars: 100 μm. (C) Comparative western blot analysis of the myogenic differentiation markers MyoG and MyoHC and the master transcription factor MyoD in the Parp3^WT^, Parp3^KO1^-FLAG, Parp3^KO1^-FLAG-Parp3^WT^, and Parp3^KO1^-FLAG-Parp3^HE^ myoblasts (sc, subconfluent) and at d3 post-differentiation. Gapdh was used as a loading control. One representative experiment is shown. See also Figure S12. (D) RT-qPCR analysis of *MyoD* and *Myf5* in differentiated Parp3^WT^, Parp3^KO1^-FLAG, Parp3^KO1^-FLAG-Parp3^WT^, and Parp3^KO1^-FLAG-Parp3^HE^ cells. *36B4* mRNA was used to normalize loading variability. Data are expressed as fold change and as means (+/− S.D.) of three independent experiments relative to the Parp3^WT^ set to 1. Statistical differences were analyzed using Student’s t test. ∗∗*p* < 0.01; ∗∗∗*p* < 0.001. (E) TMRE-mitochondrial membrane potential measured in the Parp3^WT^, Parp3^KO1^-FLAG, Parp3^KO1^-FLAG-Parp3^WT^, and Parp3^KO1^-FLAG-Parp3^HE^ cell lines at day 1 post-differentiation. Histogram represents the average quantification (+/− S.D.) of TMRE signal of three independent experiments relative to the Parp3^WT^ set to 1. Statistical differences were analyzed using Student’s t test. ∗∗*p* < 0.01; ∗∗∗*p* < 0.001.

To evaluate the functional importance of Parp3’s catalytic activity and exclude the potential off-target effects of the Crispr/Cas9 system, we re-expressed FLAG-Parp3^WT^, a catalytic dead mutant (FLAG-Parp3^HE^) or the FLAG peptide alone as control in the Parp3^KO1^ C2C12 myoblasts. We selected the restored cells for efficient expression of FLAG-Parp3^WT^ and FLAG-Parp3^HE^ (Figures 6A and S11). As expected, Parp3^KO1^-Flag displayed impaired capacity to differentiate to myotubes (Figure 6B) and confirmed reduced expression of MyoHC, MyoG, and MyoD (Figure 6C). A partial rescue of myogenic differentiation was obtained in the Parp3^KO1^-FLAG-Parp3^WT^ with the reappearance of multinucleated myotubes (Figure 6B), and to a certain extent, a restored expression of MyoHC, MyoG, and MyoD (Figures 6C and S12). That the rescue is only partial can be explained by the higher expression levels of FLAG-Parp3^WT^ compared to the endogenous levels of Parp3 or by an apparent lower functionality of the heterologous expression of the fusion protein *in cellulo*. Though the functionality of FLAG-Parp3^WT^ was previously verified *in vitro.*^20^ In contrast no rescue was detected with the FLAG-Parp3^HE^ mutant (Figures 6B, 6C, and S11). Consistently, the loss of the transcriptional expression of the two myogenic regulator factors *MyoD* and *Myf5* detected in the Parp3^KO1^-FLAG was relatively restored upon re-expression of FLAG-Parp3^WT^ but not with the FLAG-Parp3^HE^ mutant (Figure 6D). Also the reduced mitochondrial activity measured by the TMRE mitochondrial membrane potential was strengthened in the Parp3^KO1^-FLAG-Parp3^WT^ but not in the Parp3^KO1^-FLAG-Parp3^HE^ (Figure 6E). Together, these data emphasized the key contribution of Parp3’s catalytic activity in myogenic differentiation.

### Parp3 controls the abundance of H3K27 trimethylation and Ezh2 onto the promoters of selected genes

**Figure 7:**
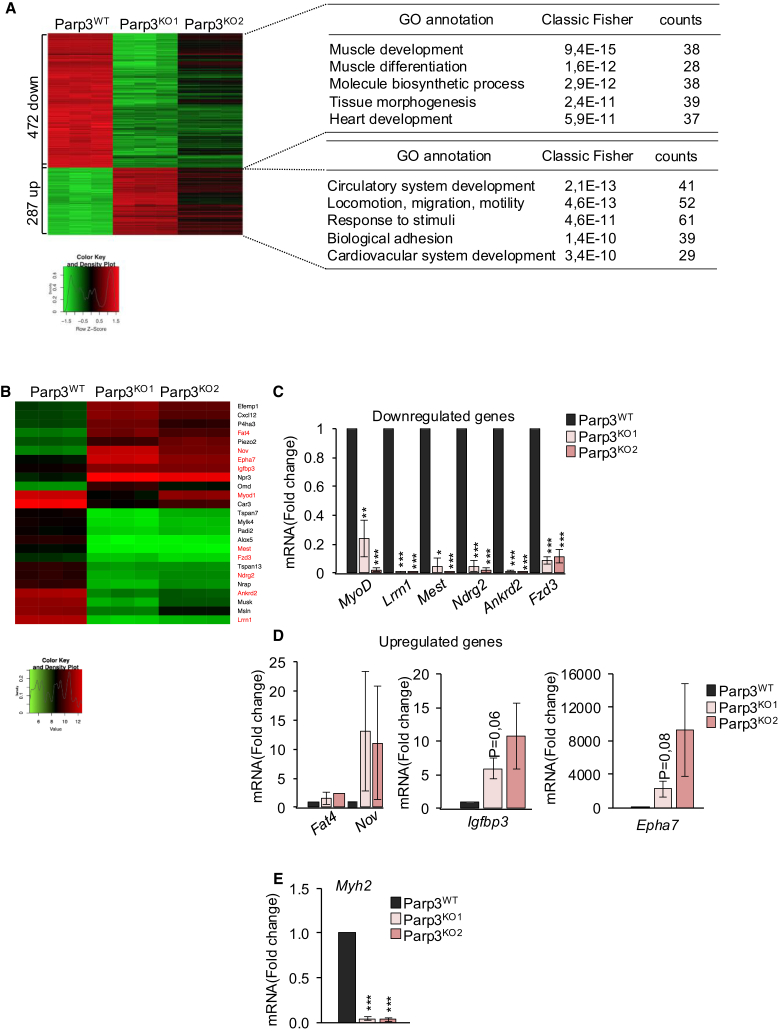
Microarray reveals dysregulation of transcripts clustering in muscle function and muscle cell dynamics in the Parp3-knockout C2C12 at day 3 post-differentiation (A) Microarray heatmap illustrating the 759 deregulated transcripts. Gene ontology (GO) analysis reveals that the down regulated genes (472) cluster in muscle development and function pathways and the upregulated genes (287) cluster in muscle cell dynamics (adhesion, mobility, response to stimuli). For each genotype, three replicates are shown. (B) Clustered heatmap of the Parp3-sensitive hit transcripts involved in muscle function and muscle cell dynamics. For each genotype, three replicates are shown. Transcripts in red were used for subsequent validation experiments. (C and D) RT-qPCR validation of selected top hit transcripts in the Parp3^WT^ versus the Parp3^KO1^ and Parp3^KO2^ cells at day 3 post-differentiation. *β*2 *microglobuline* mRNA was used to normalize loading variability. For *Igfbp3* and *Epha7*, 18S mRNA was used. Histograms represent the mRNA fold change relative to the Parp3^WT^ set to 1. Values represent the mean (+/− S.D.) of three independent experiments. Statistical differences were analyzed using Student’s t test ∗*p* < 0.05; ∗∗*p* < 0.01; ∗∗∗*p* < 0.001. (E) Relative mRNA levels of *Myh2* in differentiated Parp3^WT^, Parp3^KO1^, and Parp3^KO2^ cells. 18S mRNA was used to normalize loading variability. Histograms represent the mRNA fold change relative to the Parp3^WT^ set to 1. Values represent the mean (+/− S.D.) of four independent experiments. Statistical differences were analyzed using Student’s t test ∗∗∗*p* < 0.001.

To determine mechanisms by which Parp3 supports skeletal myogenesis, we first compared the transcriptomic profile of the Parp3^WT^ versus the Parp3^KO1^ and Parp3^KO2^ C2C12 at d3 post-differentiation (Figure 7). Differential expression analysis revealed that the abundance of a total of 759 transcripts were sensitive to the absence of Parp3. A pathway overrepresentation analysis of these genes using the GO interface revealed that the 472 downregulated genes clustered in muscle development-related pathways that are consistent with the differentiation defect. The upregulated genes were associated with mechanistic properties of the muscle cell, such as mobility, adhesion, and response to stimuli (Figure 7A). A clustered heatmap of a panel of selected hits revealed the consistent dysregulation of the genes from these groups in three biological replicates (Figure 7B). To further validate these findings, we verified the abundance of selected top hits from each group in independent differentiation experiments by qPCR. We confirmed the significant repression of *MyoD* as identified in Parp3^−/−^ TA and Parp3^−/−^ satellite-derived myotubes, we verified the repression of *Lrrn1*, *Mest*, *Ndrg2*, *Ankrd2*, and *Fzd3* coding for proteins important for muscle development (Figure 7C) and we validated a reproducible upregulation of *Nov*, *Fat4*, *Igfbp3*, and *Epha7* coding for proteins involved in response to insulin and/or adhesion events (Figure 7D). To strengthen the results in mice, we also verified the expression of *Myh2* and confirmed its significant repression in both Parp3^KO^ C2C12 clones at day 3 post-differentiation (Figure 7E).

**Figure 8:**
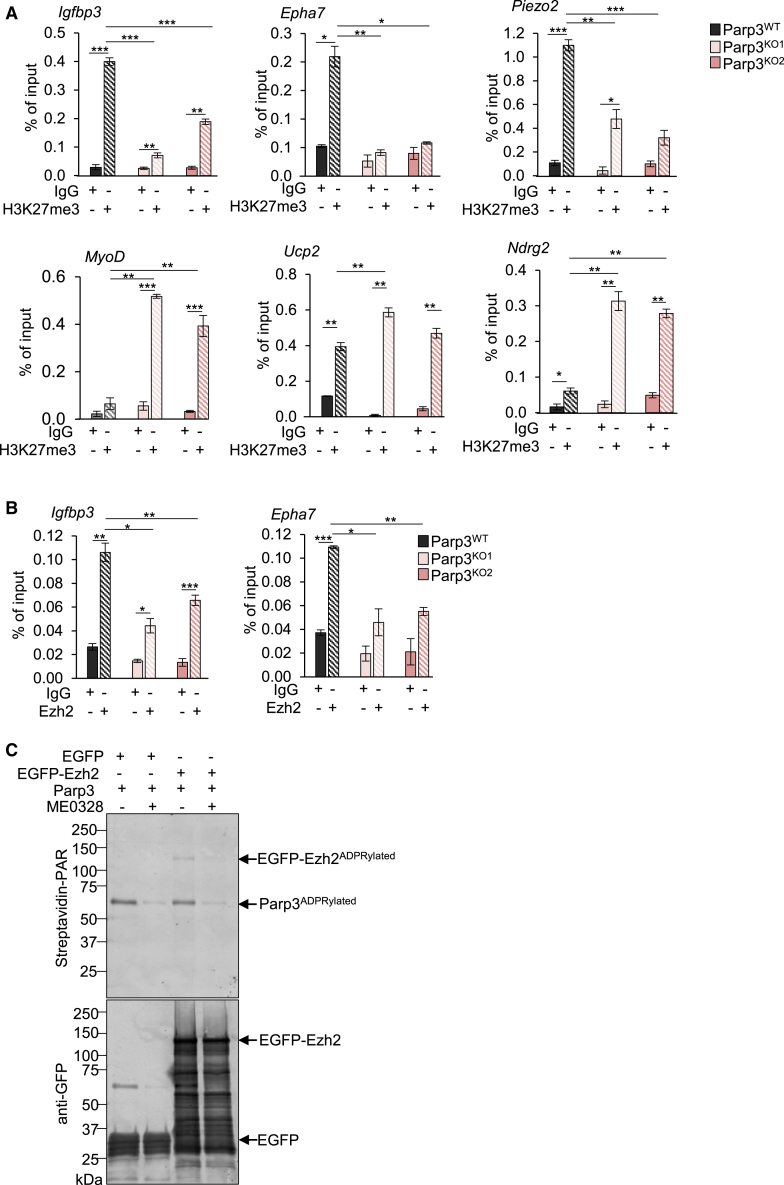
Parp3 controls the deposition of H3K27 trimethylation and Ezh2 at a subset of genes and ADP-ribosylates Ezh2 *in vitro* (A) ChIP-qPCR analyses for H3K27me3 enrichment at the promoters of *Igfbp3*, *Epha7*, *Piezo2* (upregulated in the Parp3^KO^, Figure 7B) and *MyoD*, *Ucp2*, *Ndrg2* (downregulated in the Parp3^KO^, Figure 7B) in Parp3^WT^ versus the Parp3^KO1^ and Parp3^KO2^ cells at day 3 post-differentiation. (B) ChIP-qPCR analyses for Ezh2 binding at the promoters of *Igfbp3* and *Epha7* in Parp3^WT^ versus the Parp3^KO1^ and Parp3^KO2^ cells at day 3 post-differentiation. IgG were used as ChIP negative control. Data are represented as percent of input and as mean (+/− S.D.). Statistical differences were analyzed using Student’s t test ∗*p* < 0.05; ∗∗*p* < 0.01; ∗∗∗*p* < 0.001. (C) Parp3 ADP-ribosylates Ezh2 *in vitro*. Immunopurified EGFP (control) or EGFP-Ezh2 were incubated with purified Parp3 in the presence of DNAse-activated DNA and biotinylated NAD^+^. When indicated ME0328 (20 μM) was added in the reaction buffer to inhibit Parp3 catalytic activity. ADP-ribosylated Ezh2 and Parp3 (auto-ADP-ribosylation) were detected using the streptavidin Alexa system. EGFP and EGFP-Ezh2 were further revealed by western blotting using an anti-EGFP antibody. See also Figure S5.

Studies have shown that the repressive mark H3K27me3 plays an important role in C2C12 myogenic differentiation.^12^ Moreover, Ezh2 that catalyzes H3K27me3, has been described as a binding partner of Parp3^9^ and an important molecular component of adult skeletal myogenesis.^13^^,^^21^^,^^22^ Within the top dysregulated genes, *Igfbp3* and *Epha7* have been described as direct targets of EZH2 in human cells,^23^^,^^24^ while the expression of *MyoD* has been found to vary when modulating the expression of Ezh2 in myoblasts.^25^ On these basis, we surmised on a possible functional role of Parp3 in regulating the deposition of H3K27me3 on selected genes during skeletal myogenic differentiation, may be involving Ezh2. To test this hypothesis, we first analyzed the enrichment of H3K27me3 on a panel of genes selected from the clustered heatmap including *Igfbp3*, *Epha7*, and *Piezo2* for the upregulated genes, and *MyoD and Ndrg2* for the downregulated genes by chromatin immunoprecipitation (ChIP). On the side of the mitochondrial defect, we also tested *Ucp2*. We detected a loss of enrichment of H3K27me3 at the promoters of *Igfbp3*, *Epha7*, and *Piezo2* in both Parp3^KO^ C2C12 at d3 post-differentiation that is consistent with their upregulation (Figure 8A). In contrast, while H3K27me3 is not enriched onto the promoter of *MyoD* in the control Parp3^WT^ C2C12 at d3 post-differentiation as previously reported,^12^ a significant enrichment was detected in both Parp3^KO^ C2C12 in agreement with the repression of this gene in the absence of Parp3. Moreover, compared to Parp3^WT^ C2C12, we detected a stronger enrichment of H3K27me3 onto the promoters of Ndrg2 and to a lesser extent *Ucp2* in both Parp3^KO^ C2C12 that correlate with their reduced expression (Figure 8A). For *Haus5*, a gene whose expression was not affected by the absence of Parp3 in the transcriptomic dataset, a similar H3K27me3 abundance was detected in the Parp3^KO^ versus Parp3^WT^ C2C12 (Figure S5). When analyzing the binding of Ezh2, we detected a reduced enrichment of Ezh2 onto the promoters of *Igfbp3* and *Epha7* only, while the binding of Ezh2 was not detectable onto the promoters of *Piezo2*, *MyoD*, *Ucp2*, and *Ndrg2* (Figure 8B). To further analyze the functional interplay between Ezh2 and Parp3, we evaluated the ability of Parp3 to ADP-ribosylate Ezh2 (Figure 8C). Immunopurified EGFP-Ezh2 or EGFP alone used as control were incubated together with purified Parp3, biotinylated NAD^+^, and DNAse I-treated DNA to enhance Parp3 activity. As an additional control, we used the Parp3 catalytic inhibitor ME0328. We detected an efficient auto-ADP-ribosylation of Parp3 and the ADP-ribosylation of EGFP-Ezh2 but not EGFP. Both modifications were strongly reduced in the presence of ME0328 thus identifying Ezh2 as a specific target of Parp3 catalytic activity *in vitro*. However, attempts to validate the Parp3-catalyzed ADP-ribosylation of Ezh2 *in vivo* were unsuccessful due to technical issues.

Taken together, these findings indicate that in C2C12, Parp3 regulates the abundance of H3K27me3 in a specific manner on selected genes. While Parp3 fosters H3K27me3 onto *Igfbp3 Epha7* and *Piezo2* to restrain their expression, it restricts the abundance of H3K27me3 onto *MyoD*, *Ucp2*, and *Ankrd2* to promote their expression. Moreover for *Igfbp3* and *Epha7*, the Parp3-dependent enrichment of H3K27me3 involves Ezh2.

## Discussion

Despite an increasing interest in Parp3, very little is known on its physiological functions. Moreover, beside increased sensitivity to ionizing-radiation-induced DNA damage in a Parp1-deficient background,^1^ no apparent phenotypic abnormalities have been identified in Parp3-deficient mice. Upon deeper examination, we uncovered an important role of Parp3 in astrocytic differentiation with implications in the striatum of post-natal mice both in normal conditions and upon ischemia-hypoxia providing the first demonstration for a role of Parp3 in stem cell differentiation in a physiological context.^10^

Consistently, in this study, we highlight a contribution of Parp3 in skeletal myoblast differentiation that possibly affects muscle integrity.

In the first instance, when taking the results broadly, the phenotype in the Parp3^−/−^ TA or in the Parp3^−/−^ satellite cells appeared modest compared to the strong phenotype of impaired differentiation detected in the *in vitro* stem cell based C2C12 model. Indeed, the deep analysis of TA muscle integrity revealed muscle fragility in Parp3-deficient mice that manifests by reduced muscle strength, the appearance of centrally nucleated myofibers and a reproducible though not significant increase in the expression levels of the ongoing regeneration-related markers indicating a phenotype of continuous regeneration. That these alterations were either not aggravated or not detected in older mice can be explained by the progressive age-related decline in muscle integrity that negates the phenotype. Similarly, myosatellite cells isolated from Parp3^−/−^ mice maintain an ability to differentiate as shown by the wild-type like expression of MyoG and MyoHC throughout differentiation. In contrast, the genetic loss of Parp3 or the re-expression of a catalytically inactive mutant in the *in vitro* stem cell-based myogenic model C2C12 abrogated their capacity to form myotubes. How to explain these divergences ? Likely, the contribution of Parp3 in muscle function seems compensated for *in vivo*. A possible candidate is Parp1. However, while several studies have involved Parp1 in skeletal muscle differentiation and function *in vitro*,^26^^,^^27^^,^^28^^,^^29^ a possible contribution of Parp1 to muscle integrity *in vivo* has not yet been recognized. Similarly, while Tankyrase 1 (PARP5a) has been shown to drive myogenesis *in vitro* in the C2C12 model,^30^ its role *in vivo* has not been explored. Moreover, we detected a comparable profile of PAR formation in Parp3^+/+^ and Parp3^−/−^ TA (Figure S6).

In the second instance, a more detailed examination revealed impaired expression of *MyoD* and Myh2 *in vivo* in TA that were reproduced in both *in vitro* models. Indeed, aging revealed the appearance of reduced *MyoD* expression in Parp3-deficient TA. Diminished expression of *MyoD* was reproduced in Parp3^−/−^ myosatellite cells upon differentiation and in Parp3^KO^ C2C12 myotubes. In C2C12, the mechanism involves an enhanced binding of H3K27me3 at the promoter of *MyoD*. Together, these findings suggest a role of Parp3 in the transcriptional regulation of *MyoD* both *in vivo* in skeletal muscle and *in vitro* throughout differentiation involving an epigenetic mechanism. It is known that MyoD and MyoG participate in a feedforward regulatory network, in which MyoD activates the expression of *MyoG*, and MyoG subsequently cooperates with MyoD to activate other genes i.e., *MHC*.^31^^,^^32^ Yet, while in the C2C12 model, the concomitant important reduced expression levels of MyoD, MyoG, and MHC are consistent with this model, in satellites, the reduced expression of MyoD is not associated with reduced expression of MyoG and MHC that could be explained by the milder phenotype in this model for which the remaining expression of MyoD in the Parp3^−/−^ satellites is sufficient to activate MyoG and MHC. Accordingly, in MyoD^−/−^ satellites isolated from adult skeletal muscles, only a reduced expression of MyoG has been observed.^33^

Noteworthy, young Parp3^−/−^ TA also displayed a modified composition in the rapidly contracting type II fibers compared to Parp3^+/+^ TA with a loss of the type IIA and type IIB and a compensating gain in the type IIX fibers. Consistently, a slight decrease in *Myh2* expression that encodes the fast type IIA myosin heavy chain was detected in Parp3^−/−^ myosatellite cells upon differentiation, and confirmed in Parp3^KO^ myotubes. Though a direct causal relationship with muscle weakness cannot be made here, dominant as well as recessive mutations in *MYH2* with impaired expression of MyHC-IIA have previously been associated with mild to moderate muscle weakness and internalized nuclei in humans.^34^^,^^35^^,^^36^ How does Parp3 selectively regulate the expression of *Myh2*? No data of H3K27me3 enrichment was found for the *Myh2* gene in the ChIP-Atlas database. In the zebrafish, MyoD has been shown to influence muscle fiber-type gene expression promoting the fast type muscle gene program.^37^ In human amnion-derived cells, forced expression of MyoD was shown to enhance the expression of Myh2.^38^ Knowing this, it is tempting to speculate that Parp3 participates in fast-twitch fiber type specification and promotes the expression of *Myh2* through *MyoD*. Still this hypothesis needs further investigation to formally conclude.

From the perspective of the molecular mechanism involved, we show that Parp3 tunes the enrichment of the repressive mark H3K27me3 onto a subset of genes in a binary manner in a way to supervise their expression. While for the upregulated genes *Igfbp3* and *Epha7*, the decreased enrichment of H3K27me3 detected in the Parp3^KO^ myocytes was correlated with a decreased binding of Ezh2, no efficient binding of Ezh2 could be detected on the other selected genes in the similar experimental settings. Combined with the Parp3-catalyzed ADP-ribosylation of Ezh2 identified in this study and the association of PARP3 with EZH2 previously identified in human cells,^9^ these findings suggest that in committed cells, the pro-myogenic function of Parp3 at least partly involves a gene-specific fine-tuned crosstalk with Ezh2 that remains to be decrypted. Alternatively, for another subset of genes studied here i.e., *Piezo 2*, *MyoD*, *Ucp2*, *Ndrg2*, the Parp3-regulated deposition of H3K27me3 likely involves another mechanism. Several interpretations can be proposed. A first interesting candidate is the histone lysine methyltransferase G9a. Hence, G9a has been described to catalyze H3K27me3,^39^^,^^40^ and we have previously shown that Parp3 interacts with and supervises G9a-mediated repression of selected genes.^7^ Moreover, several studies report a role of G9a in skeletal myogenesis.^41^^,^^42^^,^^43^ An other back-up mechanism could be PRC1 that has been found to stabilize H3K27me3, independently of PRC2 activity in cells undergoing skeletal muscle differentiation.^12^ At last but not least, impaired enrichment of H3K27me3 in Parp3^KO^ C2C12 can also be assigned to altered H3K27me3 demethylation. Interesting candidates would be the H3K27 demethylase KDM6A (UTX) that has been described to remove H3K27me3 marks at muscle-specific genes during myogenesis^15^ and the histone chaperone Spt6 that has been documented to coordinate H3K27me3 demethylation in coordination with KDM6A and PolII including onto MyoD.^17^ Moreover, while the mechanistic insights have been investigated here using top hit genes selected from the transcriptomic data, whether Parp3 similarly modulates the H3K27me3 deposition onto the regeneration markers remains an opened possibility. Collectively, these findings suggest a broader role of Parp3 during skeletal myogenesis not solely in interplay with Ezh2 by regulating the expression of several other genes but more largely by ADP-ribosylating and/or regulating several other proteins that remain to be identified.

Another observation in this work relies on the stress-induced skeletal muscle regeneration phenotype. We explored this question using an established local traumatic cold-induced injury model. With this model, young Parp3^−/−^ mice displayed a perturbed dynamic in the temporal expression of the embryonic-type contractile genes in TA that likely stems from the initial ongoing regeneration phenotype detected in the naive animals. Although this underlying alteration had again no obvious consequence on the overall muscle regeneration process induced by a single acute bout of injury, it is fair to posit that it can exacerbate the repair defect of Parp3^−/−^ mice under a chronic workload as enforced by long-term resistance exercise training. Moreover, when the local traumatic cold-induced injury model was applied on older individuals, the alteration in the temporal profile of regeneration markers was not observed anymore, again explained by an erasure of the phenotype when muscles wane.

Considering the *in vitro* C2C12 model, it is also interesting to emphasize that abnormal Parp3-deficient myocytes displayed alterations in the cytoskeleton organization and the accumulation of degenerative mitochondria. Parp3 has not yet been directly identified to target and/or regulate structural components of neither the cytoskeleton nor mitochondrial proteins. Nevertheless, previous works describing a fragility of the microtubule network in Parp3-deficient human cancer cells^7^ or dysfunctional mitochondria in Parp3-deficient astrocytes^10^ did indirectly reveal a connection with both entities. Moreover, in many cases and pathologies, mutations or disruption of structural or functional proteins that damage the cytoskeleton have been associated with deficiencies in mitochondrial function.^44^ These findings call attention to a possible regulatory function of Parp3 on the integrity and/or dynamic of the cytoskeleton likely in a catalytic-dependent manner, that deserves further consideration.

In summary, the data presented here point out to a key role of Parp3 and its catalytic activity in skeletal muscle differentiation and muscle integrity in mice. These results add to a recent report describing selective roles of Parp3 in differentiation events in a physiological context.^10^ Though for the differentiation of murine neural stem progenitor cells to astrocytes, the molecular scheme was different, a similar biochemical scenario involving an interplay between Parp3 and components of the PRC2 complex was proposed during neural crest specification in the zebrafish^8^ thus suggesting that Parp3 cooperates with Ezh2 to orchestrate differentiation in at the minimum two different cell lineages.

Additionally, while the selective inhibition of Parp3 emerges as a strong therapeutic option to target highly aggressive cancers, potential negative side effects onto physiological functions implicating differentiation events such as skeletal muscle integrity and/or regeneration as well as neuronal activity needs to be carefully considered.

### Limitations of the study

A first limitation concerns the mouse model used. To examine the function of Parp3 in skeletal muscle function, we utilized the constitutive Parp3 knockout mouse line. The phenotype in the Parp3^−/−^ TA or in the Parp3^−/−^ satellite cells is modest compared to the strong phenotype of impaired differentiation detected in the *in vitro* stem cell-based C2C12 model. Conditional gene targeting approaches would control any compensatory or confounding adaptations that may arise during embryonic development or across lifetime in Parp3^−/−^ mice. A second limitation has to do with the molecular mechanism explored. While Parp3 seems to control the deposition of H3K27me3 on a subset of genes, its functional interplay with Ezh2 seems limited to a few of them suggesting back-up mechanisms. A deeper examination of the chromatin landscape by comparing the genome-wide location of H3K27me3, Ezh2, and G9a in Parp3^+/+^ versus Parp3^−/−^ muscle cells would permit to further decipher the impact of Parp3 in the genome-wide remodeling of H3K27me3 in skeletal muscle differentiation. Moreover, given the documented contribution of active H3K27me3 demethylation activities in myogenesis, it would be of additional interest to explore a possible link between Parp3 and H3K27me3 demethylases or accessory proteins in the expression of key genes during myogenesis.

## Resource availability

### Lead contact

Further information and requests for resources and reagents should be directed to and will be fulfilled by the lead contact, Françoise Dantzer (francoise.dantzer@unistra.fr).

### Materials availability

The Cas9 expression plasmid generated in this study is available upon request to the lead contact.

The C2C12 Parp3^KO^ cell lines (Parp3^KO1^, Parp3^KO2^), the restored C2C12 cell lines (Parp3^KO1^-FLAG, Parp3^KO1^-FLAG-Parp3^WT^, and Parp3^KO1^-FLAG-Parp3^HE^), and the Parp3^+/+^ and Parp3^−/−^ myosatellites generated in this study are available upon request to the lead contact and with a completed materials transfer agreement.

### Data and code availability

Microarray data have been deposited at GEO and are publicly available as of the date of publication. Accession number Database: GSE255164 is also listed in the key resources table. Original/initial western blot images have been deposited at Mendeley Data: https://doi.org/10.17632/m7ckbxtbns.2 and are publicly available as of the date of publication.

All data reported in this paper will be shared by the lead contact upon request.

This paper does not report original code.

Any additional information required to reanalyze the data reported in this paper is available from the lead contact upon request.

## Acknowledgments

We thank Rajikala Suganthan for technical help in breedings and support in the grip strength tests and the Genomeast Platform of IGBMC (IGBMC, Illkirch, France) for Microarray analysis. This work of the Strasbourg drug discovery and development Institute (IMS), as part of the Interdisciplinary Thematic Institute (ITI) 2021–2028 program of the 10.13039/501100003768University of Strasbourg, 10.13039/501100004794CNRS and 10.13039/501100001677Inserm, was supported by IdEx Unistra (ANR-10-IDEX-0002) and by SFRI-STRAT’US project (ANR-20-SFRI-0012) under the framework of the French Investments for the Future Program. This work was also funded by 10.13039/501100004923AFM-Téléthon.

## Author contributions

F.D. proposed the hypotheses, conceived and supervised the study, performed experiments, and analyzed data; Z.Y., A.N., K.M.-H., N.H., N.M., I.R., and G.H. designed and performed experiments and analyzed data; J.-C.A. analyzed data; B.R.S.M., M.B., and F.D. interpreted data; F.D. wrote the paper; All authors read, revised, and approved the final manuscript.

## Declaration of interests

The authors declare no competing interests.

## STAR★Methods

### Key resources table

**Table:** 

REAGENT or RESOURCE	SOURCE	IDENTIFIER
**Antibodies**		

PARP3	Homemade, Rabbit pAb	SE4698
PARP1	Homemade, Rabbit pAb	EGT69
MyoHC	Millipore, Mouse mAb	Cat# 05–716; RRID:AB_309930
MyoHC	R and D Systems, Mouse mAb	Cat# MAB4470; RRID:AB_1293549
MyoG	Santa Cruz Biotechnology, Mouse mAb	Cat# sc-12732; RRID:AB_627980
MyoD	Invitrogen, Mouse mAb	Cat# MA1-41017; RRID:AB_2282434
GAPDH	Sigma-Aldrich, Rabbit pAb	Cat# G9545; RRID:AB_796208
Actin	Sigma-Aldrich, Rabbit pAb	Cat# A2066; RRID:AB_476693
EGFP	Roche, Mouse mAb	Cat# 11814460001; RRID:AB_390913
OXPHOS	Abcam, Mouse mAb	Cat# ab110413; RRID:AB_2629281
EZH2	Diagenode, Rabbit pAb	Cat# C15410039-50; RRID: AB_3678689
H3K27me3	Abcam, Rabbit pAb	Cat# ab192985; RRID:AB_2650559
Control IgG	Bethyl, Rabbit pAb	Cat# A90-117A; RRID:AB_67508
LC3	Nanotools, mouse mAb	Cat# 0231-100/LC3-5F10; RRID:AB_2722733
MyHC-I	DSHB, Mouse mAb	Cat# BA-D5; RRID:AB_2235587
MyHC-2A	DSHB, Mouse mAb	Cat# SC-71; RRID:AB_2147165
MyHC-2B	DSHB, Mouse mAb	Cat# BF-F3; RRID:AB_2266724
MAR/PAR E6F6A	Cell Signaling Technology, Rabbit mAb	Cat# 83732 (also 83732S); RRID:AB_2749858
Peroxidase-conjugated goat anti-Mouse	Bethyl,	Cat# A90-116P; RRID:AB_67183
Peroxidase-conjugated goat anti-Rabbit	Bethyl, Goat pAb	Cat# A120-101P; RRID:AB_67264
Peroxidase-conjugated donkey anti-Goat	Bethyl, Donkey pAb	Cat# A50-101P; RRID:AB_66755
Alexa Fluor 488 goat anti-Mouse IgG	Thermo Fisher Scientific, Goat pAb	Cat# A-11029; RRID:AB_2534088
Cyanine-conjugated goat anti-Mouse IgG2b	Jackson ImmunoResearch Labs, Goat pAb	Cat# 115-165-207; RRID:AB_2338696
Alexa Fluor 488 goat anti-Mouse IgG1	Jackson ImmunoResearch Labs, Goat pAb	Cat# 115-545-205; RRID:AB_2338854
Alexa Fluor 350 goat anti-Mouse IgM	Thermo Fisher Scientific, Goat pAb	Cat# A-31552; RRID:AB_2536169
Alexa Fluor 680 goat anti-Rabbit	Thermo Fisher Scientific, Goat pAb	Cat# A-21109; RRID:AB_2535758

**Chemicals, peptides, and recombinant proteins**		

ME0328 PARP3 inhibitor	Selleckchem	S7438; CAS: 1445251-22-8
biotinylated NAD^+^	Trevigen	Cat#4670-500-01
M.o.M solution	Abcam	Ab269452
Fluoromount-G	Invitrogen	#00-4958-02
3M Vetbond	Centravet	#14695B
DNase I-treated DNA	Enzo Life Sciences	#ALX-840-040-C010
Lipofectamine 2000	Invitrogen	#11668019
Fugene	Promega	#E2692
DMEM/GlutaMAX	Gibco	#31966
Penicillin/Streptomycin	Gibco	#15140122
Collagenase D	Roche	#11088882001
DNase1	Roche	#11284932001
RQ1 RNase-free DNase	Promega	#M6101
gelatin	Sigma	#G1393
Ham’s F12	Gibco	#21765
Ultroser	Sartorius	#15950-017
DMEM w/o sodium pyruvate	Gibco	#419650
Insulin	Sigma	#I6634
Horse serum	Hyclone	#SH30074.03
Puromycin	Gibco	A11138-03
Polybrene	Sigma	TR-1003
Bradford	Biorad	#5000006
Nitrocellulose	Amersham	#106000006
FBS	Dutscher	S1810-500
Trypsin	Life Technologies	#15090-046
HBSS buffer	Gibco	#14175095
Pefabloc	Roche	#11585916001
Protéinase K	ThermoFisher scientific	#EO0492
PIC complete mini EDTA free	Roche	#04693159001
Trizol	Ambion	#15596026
Maxima reverse Transcriptase	Thermo Scientific	#EPO0742
Protein A Sepharose	Merck Millipore	#P9424
Protein G Sepharose	Merck Millipore	#P3296
Streptavidine-Alexa Fluor 680 Conjugate	ThermoFisher	S32358
MitoSOX red reagent	Invitrogen	#M36008
GFP trap agarose	Chromotek	#GTA-20
Met2-hParp3	Amé et al. (39)	N/A

**Critical commercial assays**		

DNeasy Blood and Tissue Kit	Qiagen	#69504
TMRE assay	AbCam	Ab113852
ECL Prime detection	Cytiva	RPN2232
SYBR Green PCR kit	Qiagen	#A25776
Kapa SYBR Fast	Sigma	#KK4605
RNeasy Mini-Kit	Qiagen	#74104
Affymetrix GeneChip WT Terminal Labeling Kit	Thermo Fisher Scientific	Cat #900671
Ambion WT Expression Kit	Thermo Fisher Scientific	Cat#4411974

**Deposited data**		

Microarray	This paper	Database: GSE255164

**Experimental models: Cell lines**		

BOSC23	ATCC	Cat#CRL-11270
C2C12	Gift from Brian A. William laboratory (California Institute of technology, USA)	N/A RRID:CVCL_0188
Myosatellites	This paper	N/A

**Experimental models: Organisms/strains**		

Parp3^+/+^ and Parp3^−/−^ mice	Boehler et al. (1)	N/A
Tg:Pax7nGFP transgenic mouse line	Gift from S. Tajbakhsh (CNRS UMR3738, Institut Pasteur, Paris) (36)	Pax7nGFP/B6D2

**Oligonucleotides**		

16S-forward	Integrated DNA technologies	N/A
CCGCAAGGGAAAGATGAAAGAC		
16S-reverse	Integrated DNA technologies	N/A
TCGTTTGGTTTCGGGGTTTC		
18S-forward	Integrated DNA technologies	N/A
GCAATTATTCCCCATGAACG		
18S-reverse	Integrated DNA technologies	N/A
GGCCTCACTAAACCATCCAA		
Ankrd2-forward	Sigma Aldrich	N/A
GAGAGCCACAGAGCTCATCG		
Ankrd2-reverse	Sigma Aldrich	N/A
GCTCTTGGCCCTTAACCTTT		
Cmas-forward	Integrated DNA technologies	N/A
CAAAGGCATCCCACTGAAGA		
Cmas-reverse	Integrated DNA technologies	N/A
CCCACACACTCTGGAAGACC		
Desmin-forward	Sigma Aldrich	N/A
AAGATGGCCTTGGATGTGGA		
Desmin-reverse	Sigma Aldrich	N/A
GTTGATCCTGCTCTCCTCGC		
Epha7-forward	Integrated DNA technologies	N/A
CCGAGGAAGAGGCAGAAAA		
Epha7-reverse	Integrated DNA technologies	N/A
TGGACAACGAGAACACTGGA		
Epha7 promoter-forward	Integrated DNA technologies	N/A
CTTTGTGTAATCCGAGCACTAC		
Epha7 promoter-reverse	Integrated DNA technologies	N/A
TGCATCTTTACGACACGGTA		
Fat4-forward	Sigma Aldrich	N/A
GACAACGTCCCCACTTTTGC		
Fat4-reverse	Sigma Aldrich	N/A
AACTGCGAGTTTCCACCGAT		
Fzd3-forward	Sigma Aldrich	N/A
GATGTTTGGTGTTCCTTGG		
Fzd3-reverse	Sigma Aldrich	N/A
GCTCCTTCAGTTGGTTCT		
Haus5 promoter-forward	Integrated DNA technologies	N/A
GGGGGTCTAACAGGGCGGTGA		
Haus5 promoter-reverse	Integrated DNA technologies	N/A
GCTTGGCAACCGGTGACATCTCAG		
HK2-forward	Integrated DNA technologies	N/A
GCCAGCCTCTCCTGATTTTAGTGT		
HK2-reverse	Integrated DNA technologies	N/A
GGGAACACAAAAGACCTCTTCTGG		
Igfbp3-forward	Sigma Aldrich	N/A
AATGGCCGCGGGTTCTGC		
Igfbp3-reverse	Sigma Aldrich	N/A
TTCTGGGTGTCTGTGCTTTGAG		
Igfbp3 promoter-forward	Sigma Aldrich	N/A
GCAGATGCGTCCGCTAAAA		
Igfbp3 promoter-reverse	Sigma Aldrich	N/A
TACGTCGTTGCACTTTCCCAGCTTTCCCAG		
Lrrn1-forward	Sigma Aldrich	N/A
ACCGTTTGGCTTTCCGTAGT		
Lrrn1-reverse	Sigma Aldrich	N/A
GGATGCTGATCTCACGCAGA		
Mest-forward	Sigma Aldrich	N/A
GAAATTCAGAAGACGCTGGGTGGG		
Mest-reverse	Sigma Aldrich	N/A
CTCCAAAAACTCTGGATACG		
Mt-Co1-forward	Sigma Aldrich	N/A
ACTACCAGTGCTAGCCGCA		
Mt-Co1-reverse	Sigma Aldrich	N/A
ATCAGAACAGATGCTGGTAGA		
Myf5-forward	Sigma Aldrich	N/A
CCTGTCTGGTCCCGAAAGA		
Myf5-reverse	Sigma Aldrich	N/A
GACGTGATCCGATCCACAA		
Myh1-forward	Sigma Aldrich	N/A
ATGAACAGAAGCGCAACGTG		
Myh1-reverse	Sigma Aldrich	N/A
AGGCCTTGACCTTTGATTGC		
Myh2-forward	Sigma Aldrich	N/A
ATCCAAGTTCCGCAAGATCC		
Myh2-reverse	Sigma Aldrich	N/A
TTCGGTCATTCCACAGCATC		
Myh3-forward	Integrated DNA technologies	N/A
ACACGGATCAGAGAGCTGGA		
Myh3-reverse	Integrated DNA technologies	N/A
CCTTAACACGCCGCTCATAC		
Myh4-forward	Sigma Aldrich	N/A
AGACAGAGAGGAGCAGGAGAGTG		
Myh4-reverse	Sigma Aldrich	N/A
CTGGTGTTCTGGGTGTGGAG		
Myh8-forward	Integrated DNA technologies	N/A
CATCCACGCAGCAGATTGA		
Myh8-reverse	Integrated DNA technologies	N/A
CGCAGCAGATCACAGTCGT		
MyoD-forward	Sigma Aldrich	N/A
CTCTGATGGCATGATGGATT		
MyoD-reverse	Sigma Aldrich	N/A
GTGGAGATGCGCTCCACTAT		
MyoD promoter-forward	Integrated DNA technologies	N/A
TCAGGACCAGGACCATGTCT		
MyoD promoter-reverse	Integrated DNA technologies	N/A
CTGGACCTGTGGCCTCTTAC		
MyoZ1-forward	Integrated DNA technologies	N/A
GGAACTTGGCATTGACCTACTG		
MyoZ1-reverse	Integrated DNA technologies	N/A
AAACTTGGGCATCTGGAAGG		
MyoZ2-forward	Integrated DNA technologies	N/A
CAGAACTGCGGGATTACAGG		
MyoZ2-reverse	Integrated DNA technologies	N/A
GTCTGCCCGAAAGAGGATTG		
MyoZ3-forward	Integrated DNA technologies	N/A
TGGCAGCAGAAGTCACACTC		
MyoZ3-reverse	Integrated DNA technologies	N/A
AGTTCCAAGCCACTGAAGGAC		
ND1-forward	Integrated DNA technologies	N/A
CTAGCAGAAACAAACCGGGC		
ND1-reverse	Integrated DNA technologies	N/A
CCGGCTGCGTATTCTACGTT		
Ndrg2-forward	Sigma Aldrich	N/A
AGAACTTCGTGCGGGTTCAT		
Ndrg2-reverse	Sigma Aldrich	N/A
TCGCGACAGAATGTAGGCTC		
Ndrg2 promoter-forward	Integrated DNA technologies	N/A
GAGAAAGGAGAGGGGGGGATAGG		
Ndrg2 promoter-reverse	Integrated DNA technologies	N/A
GGGGGAAGGGGCTGGCTGAGC		
Nov-forward	Sigma Aldrich	N/A
AGATGGGCAGATTGGCTGTC		
Nov-reverse	Sigma Aldrich	N/A
ACTCCGTCGTCTGCTCAATG		
Piezo2 promoter-forward	Integrated DNA technologies	N/A
AGTAGGGCGACGCTCAGGGGTT		
Piezo2 promoter-reverse	Integrated DNA technologies	N/A
TCCTCCATCCCCTCTCCTCACC		
Ppargc1a-forward	Sigma Aldrich	N/A
GAAAGGGCCAAACAGAGAGA		
Ppargc1a-reverse	Sigma Aldrich	N/A
GTAAATCACACGGCGCTCTT		
Rpl41-forward	Integrated DNA technologies	N/A
GCCATGAGAGCGAAGTGG		
Rpl41-reverse	Integrated DNA technologies	N/A
CTCCTGCAGGCGTCGTAG		
B2-microglobuline-forward	Sigma Aldrich	N/A
CCGTCTACTGGGATCGAGAC		
B2-microglobuline-reverse	Sigma Aldrich	N/A
GCTATTTCTTTCTGCGTGCAT		
Tnni2-forward	Integrated DNA technologies	N/A
CGGAGACAGCACCTGAAGAG		
Tnni2-reverse	Integrated DNA technologies	N/A
AGACATGGAGCCTGGGATG		
Ucp2-forward	Sigma Aldrich	N/A
GAAGCTTGACCTTGGAGGC		
Ucp2-reverse	Sigma Aldrich	N/A
TACCTCCCAGAAGATGGAGA		
Ucp2 promoter-forward	Integrated DNA technologies	N/A
GCAGGGAGGGGGAGGGGAGA		
Ucp2 promoter-reverse	Integrated DNA technologies	N/A
TAGAGGGCGTGGTCGGGGCGTG		
Vimentin-forward	Integrated DNA technologies	N/A
CGGCTGCGAGAGAAATTGC		
Vimentin-reverse	Integrated DNA technologies	N/A
CCACTTTCCGTTCAAGGTCAAGC		
sgRNA targeting sequences for PARP3	This paper	N/A
GGTTCTTCGCCGCTGTTCCCT (sgRNA1)		
GGGTGTGTTGAAGCCGGCGTC (sgRNA2)		

**Recombinant DNA**		

Plasmid: pX191	This paper	N/A
Plasmid pEGFP-EZH2	Gift from Michael J Hendzel (University of Alberta, Canada)	N/A
Plasmid pEGFP	Addgene	#176015

**Software and algorithms**		

ImageJ	Schneider et al.^45^	https://imagej.net/ij/
R version 4.2.2	R Core Team (2022). R: A language and environment for statistical computing. R Foundation for Statistical Computing, Vienna, Austria.	R version 4.2.2 (2022-10-31) -- "Innocent and Trusting"Copyright (C) 2022 The R Foundation for Statistical Computing. Platform: x86_64-apple-darwin17.0 (64bit) https://www.R-project.org/.)

**Other**		

WGA AF594	Invitrogen	Cat# W11262

### Experimental model and study participant details

#### Animals

Experiments were performed in accordance with relevant guidelines and regulations. Mice were fed *ad libitum* and housed under 12h light/12h dark cycles according to the standard animal facility procedures, and experimental protocols were approved by the Ministry of Higher Education in Research and Innovation and the local ethics committee Cremeas (Comité Regional d’Ethique en Matière d’Expérimentation Animale de Strasbourg) (project APAFIS#10538). The Parp3^+/+^ and Parp3^−/−^ mice (C57BL/6J) have been described previously.^1^ The Tg:Pax7nGFP transgenic mouse line (Pax7nGFP/B6D2) was a kind gift from S. Tajbakhsh (CNRS UMR3738, Institut Pasteur, Paris).^46^ Tg:Pax7nGFP mice were crossed with Parp3^−/−^ mice producing Tg:Pax7nGFP; Parp3^+/−^ offspring that were then mate to obtain Tg:Pax7nGFP; Parp3^+/+^ and Tg:Pax7nGFP; Parp3^−/−^ for the isolation of myosatellite cells. Experiments were performed in at least three rounds and with 4 < *n* > 7 mice per round. Since no influence of the sex was observed throughout the study, both sexes of the same age were randomly assigned to experimental groups. Number of males and females for each group and per experiment are indicated in the Legends to figures. For representative images, Parp3^+/+^ and Parp3^−/−^ samples are from the same sex. Experiments were performed on 3 months-old mice (young adults) and 10 to 14 months-old mice (aged adults).

#### Cell culture

The skeletal muscle myoblast cell line C2C12 (female) was obtained from Brian A. Williams’ laboratory (California Institute of Technology, USA). C2C12 cell lines were maintained in Dulbecco’s modified Eagle’s medium (DMEM) at 4.5 g/L D-glucose, w/o sodium pyruvate (Gibco) and supplemented with 20% FBS (Dutscher), 1% P/S and incubated at 37°C and 5% CO2. C2C12 cells were not certified in-house.

Myosatellites were isolated from 3 months-old mice (2 females and 2 males/genotype), seeded on plates previously coated for 1h at 37°C with 0,02% gelatin (Sigma) and cultured under standard conditions in proliferation medium composed of 40% DMEM 4.5 g/L glucose/GlutaMAX medium (Gibco), 40% Ham’s F12 medium (Gibco) complemented with 1% P/S, 20% FBS (Dutscher), 2% Ultroser G (Sartorius) at 37°C and 5% CO2.

All cell lines are regularly tested in-house for mycoplasma contamination.

### Method details

#### Histology and immunohistochemistry on TA skeletal muscles

*Tibialis anterior* (TA) muscles were collected, immediately snap-frozen in liquid nitrogen-cooled isopentane and stored at −80°C. Haematoxylin and eosin histology analysis was performed on 10 μm thick sections according to standard protocols (Histology Department, Phenomin, ICS, Illkirch).

For immunohistochemistry, TA muscles were cut into 8 μm sections using the Leica CM3050 S cryostat, collected on glass slides, and stored at −80°C or immediately used. Monoclonal anti-myosin heavy chain (MyHC) antibodies used are listed in key resources table. Before staining, slides were allowed to thaw 1h at RT and then incubated for 1h in the dark, at RT with M.o.M solution (Abcam #ab269452) to block non-specific sites. Next, slides were stained for 1h at RT with the primary antibodies BA-D5 (MyHC-I, Type I) and SC-71 (MyHC-2A, Type 2A) diluted at 1:100 in 1X PBS, 3% BSA, washed three times for 5 min with 1X PBS, and then stained overnight at 4°C with BF-F3 (MyhHC-2B, Type 2B) diluted at 1:100 in 1X PBS, 3% BSA. Type 2X fibers remained non-stained in black. Next, slides were washed three times in 1X PBS for 5 min, and incubated for 1h with the appropriate secondary antibodies (listed in key resources table) and WGA AF594 (Invitrogen #W11262) diluted at 1:100 in 1X PBS, 3% BSA. After three washes for 5 min in 1X PBS, sections were mounted using Fluoromount-G (Invitrogen #00-4958-02) and cover slipped to dry overnight at 4°C, in the dark. Negative controls without primary antibody incubation were included to validate the specificity of the immunohistochemical staining. Images were captured using IX83 Olympus microscope equipped with an ORCA-Fusion camera (#C-14440). Single-color images were then merged to obtain a whole muscle reconstruction and quantitative analysis of the staining was performed using ImageJ.

#### Grip strength tests

Grip tests were performed to determine the maximum force displayed by the mice. Briefly, the grip strength meter was positioned horizontally and one mouse at a time was held by its tail and lowered toward the apparatus. Mice were allowed to grasp the metal grid and were then pulled backwards. The force applied to the grid just before it loses grip is recorded as the peak force. For each animal, the test was repeated 4 times leaving 10 min rest in between each measurement.

#### Freeze injury of the TA muscle

Local muscle injury was performed under isoflurane anesthesia following published protocols.^19^^,^^47^ Briefly, the TA muscle of the hindlimb was exposed by an incision though the skin. Muscle was injured by three consecutive cycles of freeze/thawing by holding firmly a metallic rod precooled in liquid nitrogen for 15 s onto the muscle and waiting 1 min for thawing before the next cycle. The skin was sutured using a surgical glue (3M Vetbond, Centravet #14695B). Mice were euthanized at d0, d4, d7 and d14 post-injury and the TA muscles were collected for analysis.

#### Isolation, culture and myogenic differentiation of satellite cells

Limb skeletal muscles were dissected from Tg:Pax7nGFP; Parp3^+/+^ and Tg:Pax7nGFP; Parp3^−/−^ mice excluding adipose tissue, nerves and tendons and were minced until turning into a slurry. Minced tissues were resuspended in 40mL DMEM 4.5 g/L glucose/GlutaMAX medium (Gibco #31966) with 1% penicillin/streptomycin (P/S, Gibco #15140122) and left 10 min to allow the separation of muscle tissues from fat tissues. Sedimented muscle tissues were resuspended in DMEM 4.5 g/L glucose/GlutaMAX medium containing 0.08% collagenase D (Roche #11088882001), 0.1% trypsin (Life Technologies #15090-046), 0.1 mg/ml of DNaseI (Roche #11284932001) and 1% P/S using 10mL/2g of dissected muscle and incubated at 37°C for 25 min in an agitating water bath (120 rpm/min) in a horizontal position. After letting the non-digested tissues settle, the supernatant was collected and filtered through 100 μm and 70μm cell strainers in 5mL FBS (Dutscher #S1810-500) per 10mL collagenase D/trypsin solution. These digestion steps were repeated until there was no more visible tissue (3–4 times). All collected supernatants were centrifuged for 10 min at 50 rcf and the pellet was discarded. Three more centrifugations were done for 15 min at 550 rcf keeping the pellet and resuspending it in 40mL DMEM 4.5 g/L glucose/GlutaMAX medium with 1% P/S. Before the last centrifugation, the cell suspension was filtered through a 40 μm cell strainer. Subsequently, the cell pellet was resuspended in 1 mL DMEM 4.5 g/L glucose/GlutaMAX medium with 1% P/S and 2% FBS.

GFP-positive cells were sorted by flow cytometry, plated at 8000 cells/cm^2^ in 48 or 24-well plates previously coated for 1h at 37°C with 0,02% gelatin (Sigma #G1393) and cultured under standard conditions in proliferation medium composed of 40% DMEM 4.5 g/L glucose/GlutaMAX medium (Gibco), 40% Ham’s F12 medium (Gibco #21765) complemented with 1% P/S, 20% FBS (Dutscher), 2% Ultroser G (Sartorius #15950-017) at 37°C and 5% CO2. Passages were made according to the proliferation rate of each primary cell line, avoiding cell-cell contact. Spontaneous immortalization was obtained by serial passaging. To induce differentiation, cells were plated in gelatin-coated 6-well plates, grown to confluency and shifted to differentiation medium composed of 50% DMEM 4.5 g/L glucose/GlutaMAX medium, 50% Ham’s F12 medium complemented with 1% P/S, 2% heat-inactivated horse serum. Differentiation medium was changed every other day until reaching the required differentiation time.

#### C2C12 myogenic differentiation

For the maintenance of proliferating myoblasts, passages were made every 2 days to avoid cell-cell contact. To induce differentiation, cells were grown to confluency and shifted to differentiation medium composed of DMEM 4.5 g/L D-glucose, w/o sodium pyruvate (Gibco #419650) supplemented with 2% heat-inactivated horse serum (Hyclone #SH30074.03), 1% P/S and 1 μM insulin (Sigma #I6634). Differentiation medium was changed every other day until reaching the required differentiation time.

#### Knockout of Parp3 using CRISPR/Cas9-mediated genome editing and generation of Parp3-rescued cell lines

Knockout cell lines were generated by transfection using Lipofectamine 2000 (Invitrogen #11668019) according to the manufacturer’s instructions with pX191, a plasmid co-expressing 2 sgRNAs targeting exon 2 and exon 6 and Cas9-EGFP under the control of the CMV promoter and bearing a Puromycin selection cassette (Figure S1). gRNA1: GGTTCTTCGCCGCTGTTCCCT (for Exon 2), gRNA2: GGGTGTGTTGAAGCCGGCGTC (for Exon 6). Two days after transfection, EGFP^+^ cells were sorted by flow cytometry and selected for three days in medium containing 1.5 μg/mL Puromycin (Gibco #A1113803). Single cells were isolated in 96 well plates, amplified and verified by sequencing and western blotting for the absence of Parp3. Two Parp3 knockout clones were selected.

Parp3^KO1^-C2C12 cells stably expressing either Flag, the catalytically active Flag-Parp3^WT^ or the dead mutant Flag-Parp3^HE^ were engineered by retroviral infection. BOSC23 cells were cotransfected with 4 μg/μL of pMX-PIE (encoding Flag, Flag-Parp3^WT^ or Flag-Parp3^HE^, described in (19)) and 0.9 μg/μL of pCL-Ecotropic plasmid using Fugene 6 (Promega #E2692). Two days later, retroviral supernatants were added to C2C12 Parp3^KO1^ cells in the presence of Polybrene (10 μg/mL, Sigma #TR-1003), centrifuged at 2500 rpm for 90 min at room temperature and incubated over night at 37°C. Selection was applied for two days with 1.5 μg/mL Puromycin followed by one week with 0.8 μg/mL Puromycin. Low-expressing EGFP^+^ cells were sorted by flow cytometry and amplified. The expression of Flag-Parp3^WT^ and Flag-Parp3^HE^ was verified by western blotting.

#### MitoSOX detection of mitochondrial ROS

C2C12 cells were seeded daily in 6-well plates to ensure that the proliferative cells and each differentiation time reached the expected time on the day of measurement. Cells were then incubated for 10 min at 37°C with 3 μM of MitoSOX Red reagent (Invitrogen #M36008) and rinsed twice with pre-warmed HBSS buffer (Gibco #14175095). Live cell fluorescence images were captured using the Typhoon FLA 9500 laser scanner (excitation: 540 nm, emission: 580 nm) and analyzed using ImageJ software. Fluorescence measured in the differentiated cells was represented as a ratio of fluorescence detected in the sub-confluent cells.

#### Mitochondrial (mt)DNA versus nuclear (n)DNA ratio

The mtDNA copy number was analyzed by qPCR according to Quiros et al.^48^ Briefly, DNA from proliferative and differentiated C2C12 or satellite cells was isolated using “DNeasy Blood and Tissue Kit” (Qiagen #69504). The mitochondrial-specific target genes selected for our assay were NADH dehydrogenase 1 (ND1) and the ribosomal ARN 16S while the nuclear-specific target gene was the hexokinase 2 (HK2). The primer sequences are listed in key resources table. Analysis of the mtDNA/nDNA ratio was calculated by following the classical ΔΔCt method used for qPCR analysis.

#### TMRE mitochondrial membrane potential assay

The evaluation of the mitochondrial membrane potential was performed using the TMRE mitochondrial membrane potential assay (AbCam #ab113852) kit following the manufacturer’s instructions. Briefly, C2C12 cells were seeded in 6-well plates and either grown to sub-confluency or differentiated for 24h. Cells were then stained for 20 min with 50 μM TMRE added to the culture medium and fluorescence was captured using the Amersham Typhoon laser scanner (GE Healthcare).

#### Cell extracts and western blotting

For whole cell extracts, cells were lysed in cold RIPA-like buffer (50 mM Tris-HCl pH 8, 0.5% Triton X-100, 0.25% Sodium deoxycholate, 150 mM NaCl, 1 mM EDTA, 50 mM NaF, 20 mM Sodium pyrophosphate, 1 mM Sodium orthovanadate, 1 mM Pefabloc (Roche #11585916001) and Protease Inhibitor Cocktail (PIC) « complete mini EDTA free » (Roche #04693159001) for 15 min on ice. For TA tissue extracts, half of the muscle was collected in 400 μL RIPA-like buffer and dissociated with ceramic beads using the Precellys homogenizer (Bertin Technologies). After centrifugation at 13,000 rpm for 15 min at 4°C, clear lysates were collected. For LC3 detection, cells were lysed in cold mRIPA buffer (50 mM Tris-HCl pH 7.5, 400 mM NaCl, 1 mM EDTA, 1% NP40, 0.1% Sodium deoxycholate) and centrifuged at 13,000 rpm for 20 min at 4°C and clear lysates were collected. For nuclear extracts, cells were resuspended in cold hypotonic buffer (10 mM Tris-HCl pH 7.3, 10 mM KCl, 1.5 mM MgCl2, 10 mM β-mercaptoethanol, 0.2 mM PMSF) and homogenized on ice by using a glass Dounce homogenizer (pestle B). After centrifugation at 4000 rpm for 5 min at 4°C, nuclear pellets were resuspended in cold extraction buffer (15 mM Tris-HCl pH 7.3, 0.4 M NaCl, 1 mM MgCl2, 1 mM EDTA, 1% glycerol, 10 mM β-mercaptoethanol, 0.2 mM PMSF) and centrifuged at 12,000 rpm for 30 min at 4°C. The supernatant was used as the nuclear extract fraction. For all extracts, protein quantification was made using Bradford assay (BioRad #5000006) and equivalent amounts of proteins were dissolved in loading buffer and denatured by heating for 5 min at 95°C before being loaded onto 8% or 10% SDS–PAGE gel and separated by electrophoresis. Proteins were then transferred on a nitrocellulose membrane (Amersham #106000006) for 2h at 100 V. Membranes were blocked with 5% powdered skimmed milk in 1X TBS-0.1% Tween for 1h, followed by incubation with the indicated primary antibody O/N at 4°C (key resources table). Protein bands were visualized using ECL Prime detection system (Cytiva #RPN2232) and the images were captured using the Amersham Imager 680 (GE Healthcare Life Science) or the Odyssey DLx Imager (LI-COR Biosciences) when using fluorescent-dye conjugated antibodies.

#### Ultrastructural and immunofluorescence imaging

For ultrastructural analysis, C2C12 cells were induced to differentiate for 72h. Cells were fixed by immersion in 2.5% glutaraldehyde and 2.5% paraformaldehyde in cacodylate buffer (0.1 M, pH 7.4), washed in cacodylate buffer for further 30 min. The samples were postfixed in 1% osmium tetroxide in 0.1M cacodylate buffer for 1h at 4°C and dehydrated through graded alcohol (50, 70, 90, and 100%) and propylene oxide for 30 min each. Samples were oriented and embedded in Epon 812. Semithin sections were cut at 2 μm and ultrathin sections were cut at 70 nm (Leica Ultracut UCT) and contrasted with uranyl acetate and lead citrate and examined at 70kv with a Morgagni 268D electron microscope (FEI Electron Optics, Eindhoven, the Netherlands). Images were captured digitally by Mega View III camera (Soft Imaging System).

For immunofluorescence and fusion index, cells were grown and differentiated for the indicated time points on glass coverslips. Cells were washed twice in 1X PBS, fixed for 15 min at room temperature in 4% formaldehyde diluted in blocking buffer (1X PBS -0,1% Triton X-100-0,1% skimmed milk) and washed again three times for 10 min in blocking buffer. Cells were then incubated overnight at 4°C with the mouse anti-MyoHC antibody (1:50 Abcam) diluted in blocking buffer, washed three times for 10 min with blocking buffer and incubated for 1h at RT in Alexa Fluor 488 conjugated goat anti-mouse secondary antibody (1:1500, Thermo Fisher #A-11029). Slides were mounted with the Fluoromount-G mounting medium containing DAPI. Images were captured using a Leica TCS40D microscope (Leica Microsystems GmbH) equipped with an ORCA-ER chilled CCD camera (Hammamatsu) and the capture software Openlab (Improvision).

#### Microarray and qRT-PCR

Total RNA from C2C12 was extracted using the RNeasy Mini-Kit (Qiagen #74104) following the manufacturer’s instructions. For the TA, one-third of the muscle was collected in 600 μL TRIzol reagent (Ambion #15596026) and dissociated with ceramic beads using the Precellys homogenizer (Bertin Technologies) prior to RNA extraction.

For microarray analysis, biotinylated single strand cDNA targets were prepared, starting from 200 ng of total RNA, using the Ambion WT Expression Kit (Thermo Fisher #4411974) and the Affymetrix GeneChip WT Terminal Labeling Kit (Thermo Fisher #900671) according to Affymetrix recommendations. Following fragmentation and end-labeling, 3 μg of cDNAs were hybridized for 16 h at 45^o^C on *GeneChip Mouse Gene 2.0 ST arrays* (Affymetrix) interrogating 35240 RefSeq transcripts and ∼2000 LncRNAs represented by approximately 27 probes spread across the full length of the transcript. The chips were washed and stained in the GeneChip Fluidics Station 450 (Affymetrix) and scanned with the GeneChip Scanner 3000 7G (Affymetrix) at a resolution of 0.7 μm. Raw data (.CEL Intensity files) were extracted from the scanned images using the Affymetrix GeneChip Command Console (AGCC) version 4.1.2.

CEL files were further processed with Affymetrix Expression Console software version 1.4.1 to calculate probe set signal intensities using Robust Multi-array Average (RMA) algorithms with default settings.

For RT-qPCR, 1 μg of DNase-treated RNA (Promega #M6101) was reverse transcribed using the Maxima Reverse Transcriptase (Thermo Scientific #EPO742) according to the manufacturer’s instructions. Real-time PCR was performed using the SYBR Green PCR kit (Qiagen #A25776) or the Kapa SYBR Fast (Kapa Biosystems #KK4605) following the manufacturer’s instructions and combined with the Applied Biosystems StepOne (Life Technologies) detection system. The quantity of PCR products was determined by the 2ΔΔ-CT quantification method. All samples were analyzed in duplicates or triplicates and normalized using *β2-microglobuline* or *18S* for C2C12 cells and *Cmas*, *Rpl41* or *18S* for mice. The primer sequences used for RT-qPCR are listed in key resources table.

#### Chromatin immunoprecipitation (ChIP)

For each ChIP assay, Parp3^WT^ and Parp3^KO^ C2C12 cells were differentiated in 150 mm-cell dishes (respectively 5 and 7 plates) for 72h. Cells were fixed with 1% formaldehyde for 5 min. Fixation was quenched by adding 0.125 M of glycine for 5 min. Fixed cells were washed twice with ice-cold 1X PBS and lysed in ice-cold lysis buffer (5 mM PIPES pH 8, 85 mM KCl, 0,5% NP-40 and PIC (Roche)) for 1h on ice. Cells were then homogenized 50 times on ice using a Dounce. The crude nuclear pellet was collected by centrifugation and lysed in chromatin extraction buffer (50 mM Tris pH 8.1, 10 mM EDTA pH 8, 1% SDS and PIC (Roche)) for 1h on ice. Chromatin was sheared by sonication using the Covaris sonicator (peak power 200W, duty factor 5%, cycles/burst 250) to generate chromatin fragments (100–500 bp size). The soluble chromatin was diluted 1:5 in dilution buffer (0,01% SDS, 1% Triton X-100, 1.2 mM EDTA, 16,7 mM Tris pH 8.1, 167 mM NaCl and protease inhibitor cocktail (Roche)), pre-cleared 2h with pre-blocked protein A/G agarose beads (Millipore #P9424, #P3296) at 4°C and centrifuged at 4000 rpm for 10 min at 4°C. Input was collected (100 μL) and an equivalent amount of 50 μg of chromatin extract was then immunoprecipitated using the specific antibodies (listed in key resources table) overnight at 4°C. Immune complexes were recovered by adding 100 μL of pre-blocked agarose beads for 2h at 4°C. The immunoprecipitated material was washed once for 5 min with dialysis buffer (2 mM EDTA, 50 mM Tris pH 8.1, 0,2% N-Lauroylsarcosine sodium salt and PIC (Roche)), five times for 5 min with washing buffer (100 mM Tris pH 8.1, 500 mM LiCl, 1% NP-40, 1% sodium deoxycholate and protease inhibitor cocktail (Roche)) and twice with Tris-EDTA (TE). Beads were resuspended in 200 μL TE for samples and 100 μL for inputs. De-crosslinking was performed by adding RNAse A (50 μg/mL) for 30 min at 37°C under shaking (800 rpm) followed by 4 μL of 10% SDS and incubation overnight at 70°C under shaking (1300 rpm). The bounded DNA was eluted from the beads by adding proteinase K (ThermoFisher #EO0492, 0.2 mg/mL) at 45°C for 90 min under shaking (800 rpm). The immunoprecipitated DNA was purified, precipitated and analyzed by qPCR using the gene specific primers listed in key resources table. ChIP-qPCR results are represented as percentage of IP/input signal (% input).

#### *In vitro* PARylation

To analyze Parp3-catalysed ADP-ribosylation of Eah2, HEK 293 cells were transfected with either pEGFP-Ezh2 or pEGFP as control for 24h. Cleared RIPA-like whole cell extracts (1,5 mg) were diluted 6X in dilution buffer (15 mM TrisHCl pH 8, 150 mM NaCl) and incubated for 4h at 4°C in the presence of agarose beads coupled to GFP (Chromotek #GTA-20). After centrifugation at 5000 rpm at 4°C for 5 min, beads were washed twice in washing buffer (15 mM TrisHCl pH 8, 250 mM NaCl), and twice in (15 mM TrisHCl pH 8, 150 mM NaCl) and resuspended in 250 μL of washing buffer with 250 mM NaCl. Next, 1/10 volume of the beads were incubated for 1 h at 30°C in 40 μL of reaction buffer containing biotinylated NAD^+^ (Trevigen # 4670-500-01, 6,25 μM), DNase I-treated DNA (Enzo #ALX-840-040-C010, 2μg), purified Parp3 (Met2-hParp3, 2,8 μg^49^) and 4 μL 10X reaction buffer (50 mM TrisHCl pH8, 100 mM NaCl, 1mM DTT). The beads were then washed extensively in 1X PBS to reduce the auto-modified Parp3. Proteins were eluted by boiling in Laemmli buffer, separated on a 8–20% SDS-PAGE gel and transferred onto a nitrocellulose membrane. After blocking with 1X PBS -0,1% Tween-5% BSA, the membrane was incubated for 1h at RT with the Streptavidine-Alexa 680 (ThermoFisher #S32358, diluted at 1:30000 in 1X PBS-0,01% Tween-0,5%BSA). Images were captured using the Odyssey DLx Imager (LI-COR Biosciences). Loaded EGFP fusion proteins were verified by immunoblotting using the appropriate antibody (key resources table).

### Quantification and statistical analysis

R software was used to perform satistics. Quantitative data are presented as mean ± sd or +/− sem as indicated in the Figure legends. Comparisons between two conditions were analyzed using Student’s t test and comparisons between more conditions or groups of mice were analyzed using the Wilcoson-Mann-Withney test. A *p* value < 0.05 was considered statistically significant for all comparisons.
