# Supplementary material for: Parp3 assists muscle function and skeletal muscle differentiation by selectively adjusting H3K27me3 enrichment
Source: iScience. 2025 Mar 25;28(4):112267. doi: 10.1016/j.isci.2025.112267 (PMC12005933; doi:10.1016/j.isci.2025.112267)
Supplement: Document S1. Figures S1–S12 [file mmc1.pdf]

iScience, Volume *28*

Supplemental information

Parp3 assists muscle function and skeletal muscle differentiation by selectively adjusting H3K27me3 enrichment

Zuleyha Yildirim, Aurélia Noll, Kathline Martin-Hernandez, Jean-Christophe Amé, Najat Hanini, Nadia Messaddeq, Isabelle Robert, Bernardo Reina San Martin, Gunn Hildrestrand, Magnar Bjoras, and Françoise Dantzer

Legends to Figures

Fig. S1 (complement to Fig. 1,3). The absence of Parp3 induces TA muscle weakness but does not affect overall muscle regeneration upon injury. (a) Additional representative H&E images of TA muscles from 3 months and 10-12 months male Parp3$^{+/+}$ and Parp3$^{-/-}$ mice showing the appearance of centrally nucleated myofibers (indicated by arrows). Scale bars: 125 µm. (b) Quantification of myofiber cross sectional mean Feret's diameter distribution in TA muscles from 3 months old Parp3$^{+/+}$ and Parp3$^{-/-}$ mice. The two distribution shapes correspond to the independent quantification of the small and large fibers (n=7 mice/genotype, 4 males, 3 females). (c) Parp3$^{-/-}$ mice display grip strength deficits. Grip strength was measured in male Parp3$^{+/+}$ and Parp3$^{-/-}$ mice of various ages (3<n>7/genotype/age). Statistical differences were calculated using Student's t test *P<0,05; **P<0,01. (d) Analysis of muscle regeneration upon acute injury. *Top*, diagram of the experimental approach. Three months-old Parp3$^{+/+}$ and Parp3$^{-/-}$ mice were exposed to freeze-induced muscle injury. TA muscle biopsies were taken from non-injured control mice (naive) and at days d4, d7 and d14 post-injury for histology. *Bottom*, Representative H&E images of TA muscles throughout regeneration. The experiment shown was perfomed with females. Arrows denote centrally nucleated fibers.

Fig. S2 (complement to Fig. 3). The absence of Parp3 does not alter the profile of ongoing nor late regeneration-related genes upon acute muscle injury in old adults. (a). Diagram of the experimental approach. (b) RT-qPCR expression analysis of of *Myh3*, *Myh8*, *Desmin*, *Vimentin* regeneration-related genes in TA muscles from 14 months-old Parp3$^{+/+}$ and Parp3$^{-/-}$ mice left non-injured (ctl) and at the indicated time points throughout muscle regeneration. (c) RT-qPCR expression analysis of *Myoz1*, *Myoz2*, *Myoz3*, *Tnni2* genes associated with myofiber maturity in TA muscles from 14 months-old Parp3$^{+/+}$ and Parp3$^{-/-}$ mice left non-injured (ctl) and at the indicated time points throughout muscle regeneration. *Cmas* mRNA was used for normalization. Data are expressed as fold change (Log 10) with one individual/genotype set to one as a reference and were analysed by Wilcoxon-Mann-Whitney test *P<0,05. n= 4 mice/condition (Parp3$^{+/+}$ctl: 4 males, Parp3$^{-/-}$ctl: 1 female, 3 males, Parp3$^{+/+}$d4: 2 males, 2 females, Parp3$^{-/-}$d4: 2 males, 2 females, Parp3$^{+/+}$d7: 2 males, 2 females, Parp3$^{-/-}$ d7: 2 males, 2 females, Parp3$^{+/+}$d14: 2 males, 2 females, Parp3$^{-/-}$d14: 2 males, 2 females.

Fig. S3. (complement to Fig. 4). Generation of the Parp3^{KO} C2C12 cell lines using the CRISPR/Cas9 technology. (a) Schematic representation of the *PARP3*-targeting gRNA sequences. Guide RNAs targeting respectively exon 2 (blue) and exon 6 (red) are indicated in color. PAM, protospacer adjacent motifs are indicated in capital letter. Cutting sides of Cas9 are indicated by red arrows. (b) Schematic representation of the Cas9 expression plasmid. The all in one pX191 vector contains the two targeted gRNAs under the control of the U6 promoter, the Cas9-GFP under the control of the CMV promoter and the Puromycin selection cassette and a Poly(A) tail sequence under the control of an SV40 promoter.

Fig. S4 (complement to Fig. 5). Parp3^{KO} C2C12 display altered mitochondrial function throughout differentiation. (a) Protein expression levels of complex II, III and V (OXPHOS) were analysed by western blotting in Parp3^{WT} and Parp3^{KO} proliferating myoblasts (sc, subconfluent), confluent myoblasts (d0) and at the indicated times of differentiation. Actin was used as a loading control. A representative experiment is shown. Histograms represent the fold change relative to the sc-Parp3^{WT} set to 1. Data are expressed as means (+/- sem) of three independent experiments. Values were obtained using ImageJ software and normalized using Actin as a control. Statistical differences were calculated using Student's t test *P<0,05. (b) Protein expression levels of LC3-I and LC3-II in Parp3^{WT} and Parp3^{KO1} proliferating myoblasts

(sc, subconfluent), confluent myoblasts (d0) and at the indicated times of differentiation. Actin was used as a loading control. One representative experiment out of three is shown. The enhanced conversion of LC3-I to LC3-II detected in the $Parp3^{KO1}$ compared to $Parp3^{WT}$ differentiating cells suggest the appearance of autophagy. (c) Relative quantification of mtDNA (using qPCR of either 16S (*left*) or ND1 (*right*)) versus nDNA (using qPCR of HK2) in the $Parp3^{WT}$ versus the $Parp3^{KO1}$ and $Parp3^{KO2}$ subconfluent (sc) cells and at the indicated time points throughout myogenic differentiation. Histograms represent the fold change relative to the $Parp3^{WT}$ set to 1. Data are expressed as means (+/- sem) of four independent experiments. Statistical differences were calculated using Student's t test *p<0,05.

Fig. S5 (complement to Fig. 8) The enrichment of H3K27me3 onto the *Haus 5* gene is not impaired by the absence of Parp3. ChIP-qPCR analyses for H3K27me3 enrichment at the promoter of *Haus5* in $Parp3^{WT}$ versus the $Parp3^{KO1}$ and $Parp3^{KO2}$ C2C12 cells at day 3 post-differentiation. IgG were used as ChIP negative control. Data are represented as percent of input and as mean (+/- S.D.). Statistical differences were calculated using Student's t test *P<0,05.

Fig. S6 (complement to discussion). The absence of Parp3 does not impair the basal levels of ADP-ribosylation in TA biopsies. Total protein extracts from TA biopsies were analysed for the expression levels of Mono (MAR) and Poly(PAR) ADP-ribosylation and actin was used as loading control. The results are shown for 6 female mice/genotype.

Fig. S7 (complement to Fig.2) Original unadjusted Western blot images for Figure 2a.

Fig. S8 (complement to Fig. 4) Original unadjusted Western blot images for Figure 4a.

Fig. S9 (complement to Fig. 4) Original unadjusted Western blot images for Figure 4b.

Fig. S10 (complement to Fig. 4) Original unadjusted Western blot images for Figure 4f.

Fig. S11 (complement to Fig. 6) Original unadjusted Western blot images for Figure 6a.

Fig. S12 (complement to Fig. 6) Original unadjusted Western blot images for Figure 6c.

Fig. S1

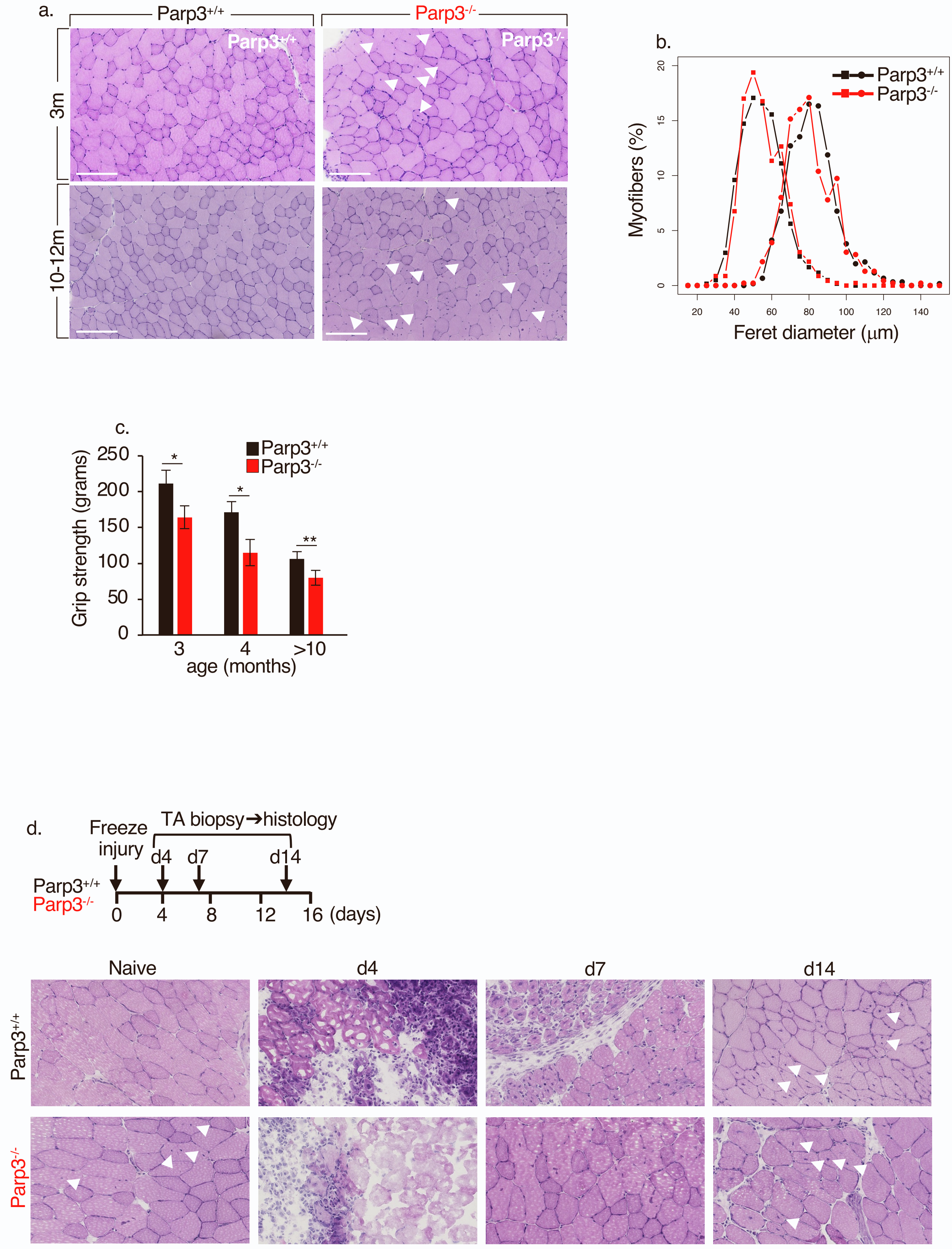
a.
Parp3+/+
Parp3-/-
3m
10-12m
b.
Parp3+/+
Parp3-/-
Myofibers (%)
Feret diameter (μm)
0
5
10
15
20
20
40
60
80
100
120
140
c.
Parp3+/+
Parp3-/-
Grip strength (grams)
250
200
150
100
50
0
*
*
**
3
4
>10
age (months)
d.
Freeze
injury
TA biopsy➔histology
d4
d7
d14
Parp3+/+
Parp3-/-
0
4
8
12
16
(days)
Naive
d4
d7
d14
Parp3+/+
Parp3-/-

Fig. S2

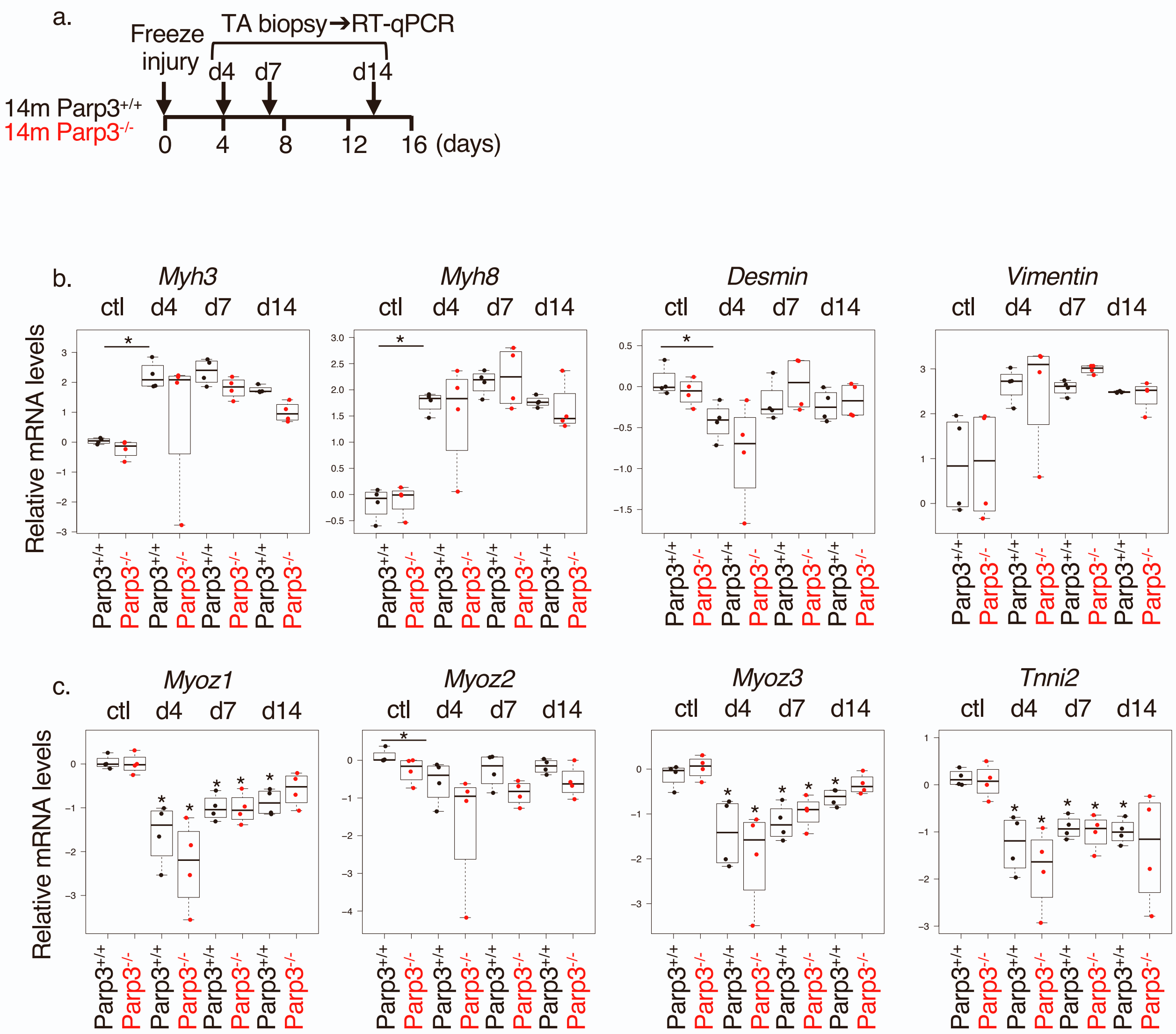

a.
Freeze injury
TA biopsy➔RT-qPCR
d4 d7 d14
14m Parp3+/+
14m Parp3-/-
0 4 8 12 16 (days)
b.
Myh3
Myh8
Desmin
Vimentin
ctl d4 d7 d14
Relative mRNA levels
Parp3+/+ Parp3-/-
c.
Myoz1
Myoz2
Myoz3
Tnni2
ctl d4 d7 d14
Relative mRNA levels
Parp3+/+ Parp3-/-

Fig. S3

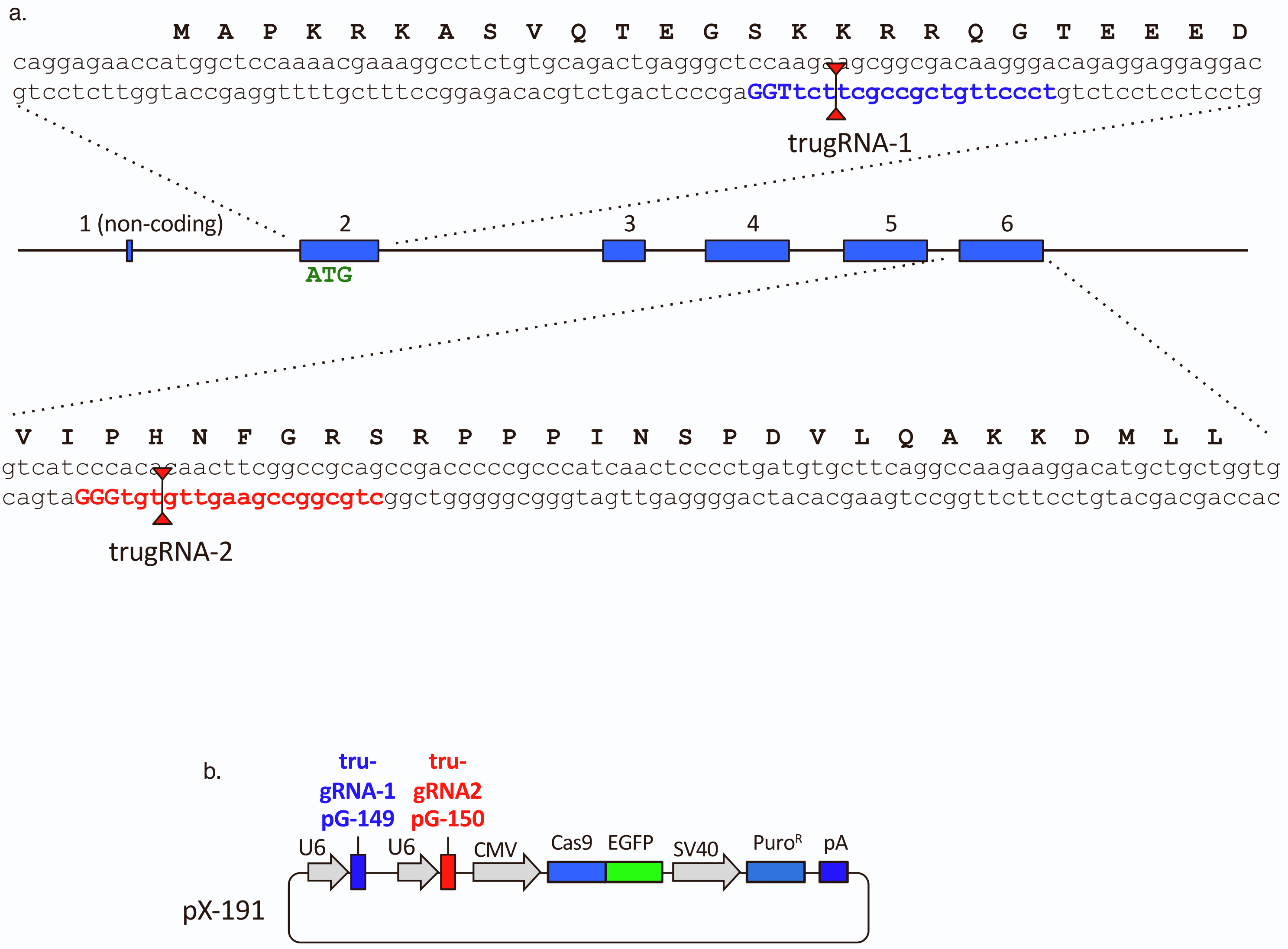
a.
M A P K R K A S V Q T E G S K K R R Q G T E E E D
caggagaaccatggctccaaaacgaaaggcctctgtgcagactgagggctccaagaagcggcgacaagggacagaggaggaggac
gtcctcttggtaccgaggttttgctttccggagacacgtctgactcccgaGGTtcttcgccgctgttccctgtctcctcctcctg
trugRNA-1
1 (non-coding)
2
3
4
5
6
ATG
V I P H N F G R S R P P P I N S P D V L Q A K K D M L L
gtcatcccacacaacttcggccgcagccgacccccgcccatcaactcccctgatgtgcttcaggccaagaaggacatgctgctggtg
cagtaGGGtgtgttgaagccggcgtcggctgggggcgggtagttgaggggactacacgaagtccggttcttcctgtacgacgaccac
trugRNA-2
b.
tru-
gRNA-1
pG-149
tru-
gRNA2
pG-150
U6
U6
CMV
Cas9
EGFP
SV40
Puro^R
pA
pX-191

Fig. S4

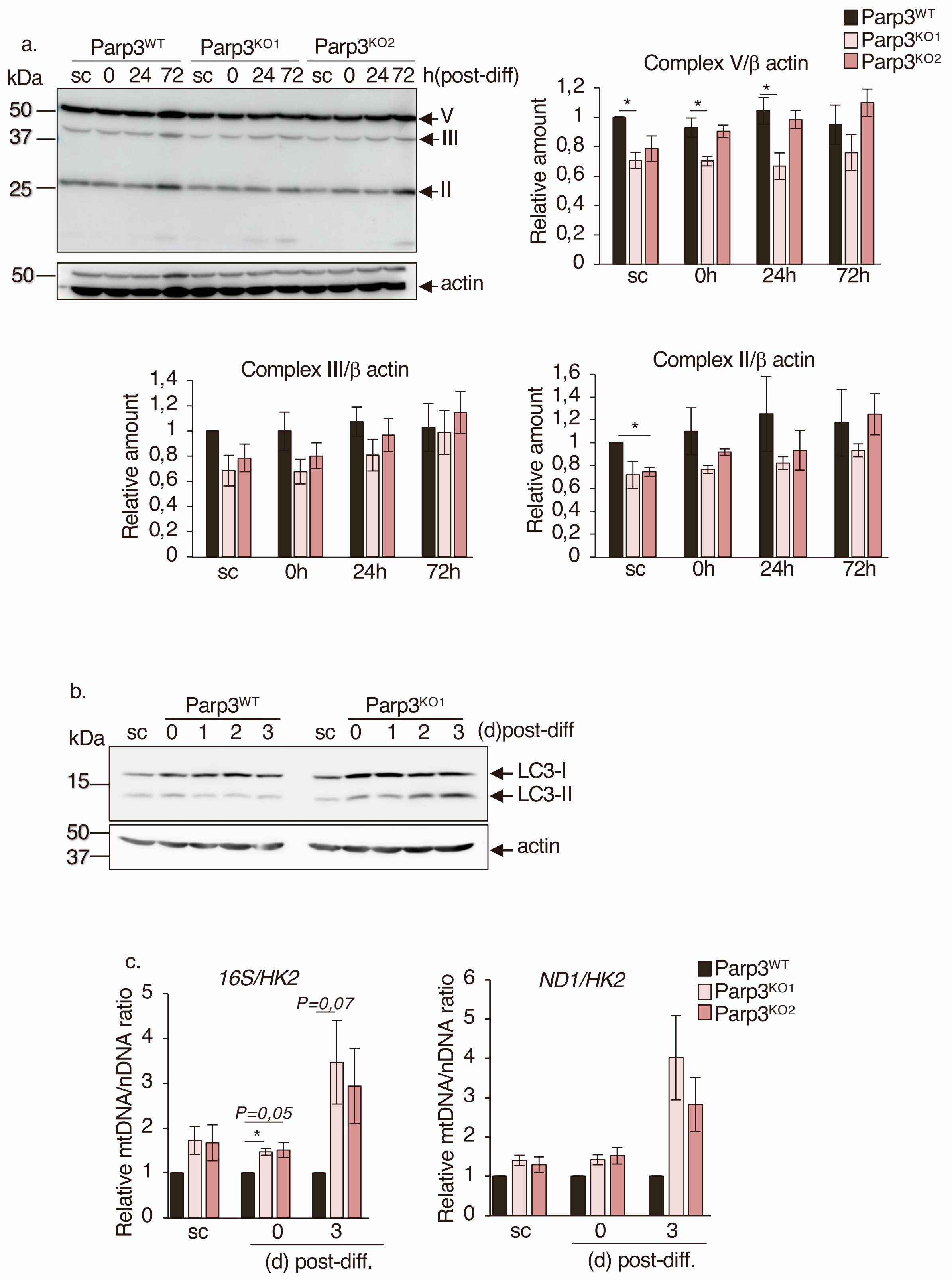

a.
Parp3WT
Parp3KO1
Parp3KO2
kDa
sc 0 24 72 sc 0 24 72 sc 0 24 72 h(post-diff)
50
37
25
V
III
II
50
actin
Complex V/β actin
Relative amount
0
0,2
0,4
0,6
0,8
1
1,2
*
sc
0h
24h
72h
Complex III/β actin
Relative amount
0
0,2
0,4
0,6
0,8
1
1,2
1,4
sc
0h
24h
72h
Complex II/β actin
Relative amount
0
0,2
0,4
0,6
0,8
1
1,2
1,4
1,6
*
sc
0h
24h
72h
b.
Parp3WT
Parp3KO1
kDa
sc 0 1 2 3 sc 0 1 2 3 (d)post-diff
15
LC3-I
LC3-II
50
37
actin
c.
16S/HK2
Relative mtDNA/nDNA ratio
0
1
2
3
4
5
P=0,05
*
P=0,07
sc
0
3
(d) post-diff.
ND1/HK2
Relative mtDNA/nDNA ratio
0
1
2
3
4
5
6
sc
0
3
(d) post-diff.
Parp3WT
Parp3KO1
Parp3KO2

Fig. S5

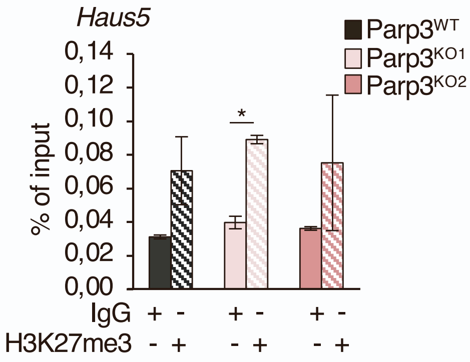

Haus5
Parp3WT
Parp3KO1
Parp3KO2
% of input
0,14
0,12
0,10
0,08
0,06
0,04
0,02
0,00
*
IgG + - + - + -
H3K27me3 - + - + - +

Fig. S6

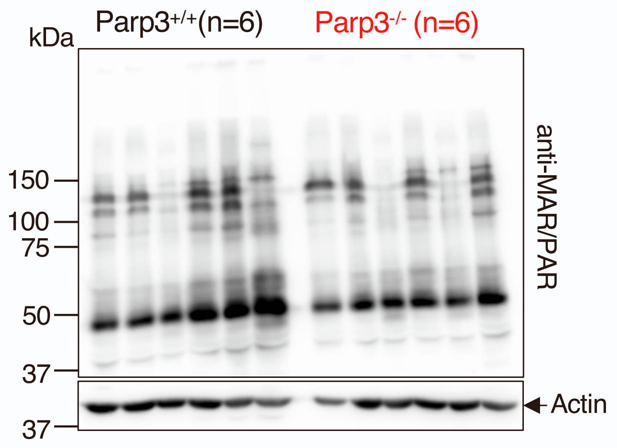

Fig. S7

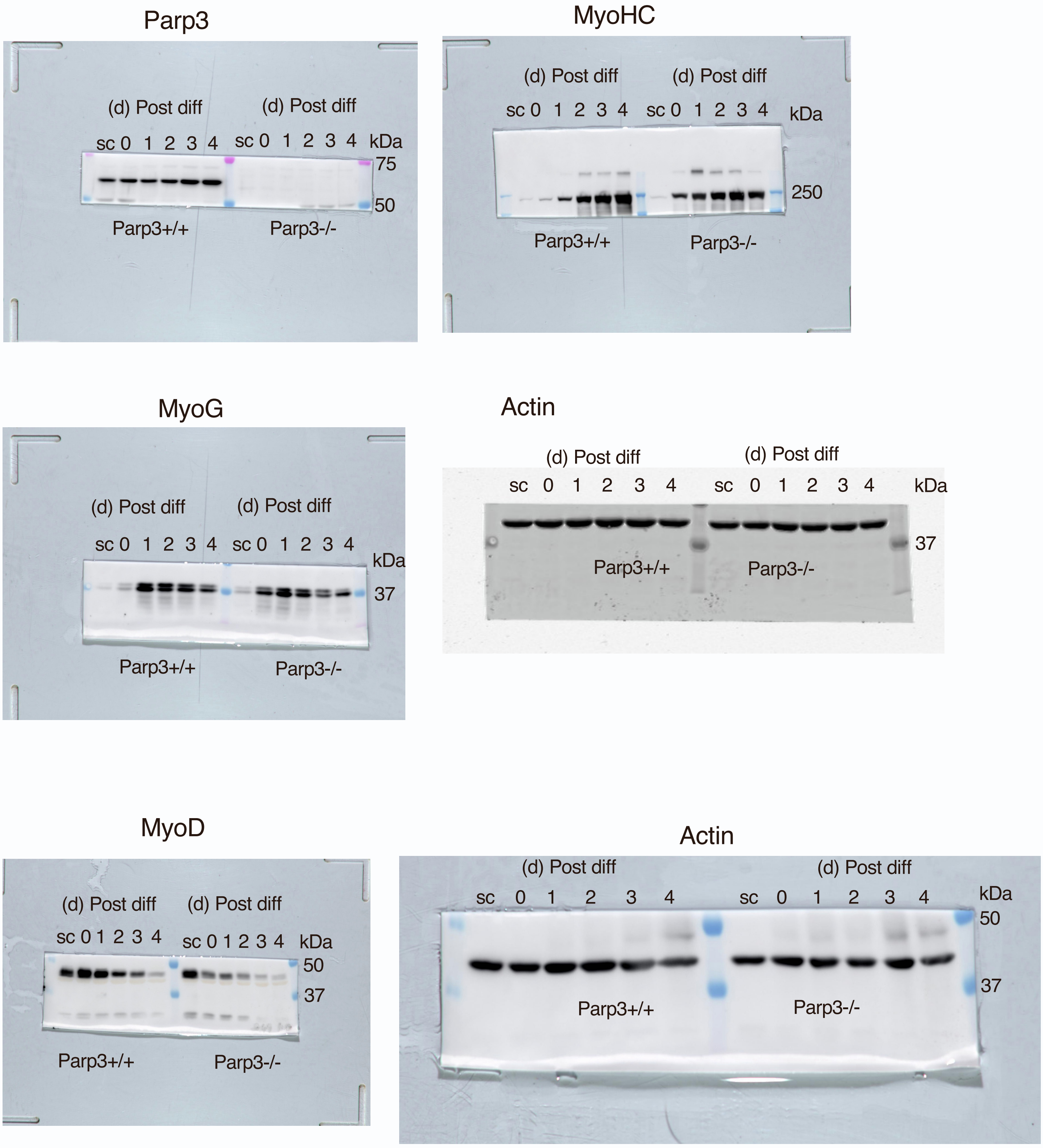

Parp3
(d) Post diff
(d) Post diff
sc 0 1 2 3 4 sc 0 1 2 3 4 kDa
75
50
Parp3+/+
Parp3-/-
MyoHC
(d) Post diff
(d) Post diff
sc 0 1 2 3 4 sc 0 1 2 3 4 kDa
250
Parp3+/+
Parp3-/-
MyoG
(d) Post diff
(d) Post diff
sc 0 1 2 3 4 sc 0 1 2 3 4
kDa
37
Parp3+/+
Parp3-/-
Actin
(d) Post diff
(d) Post diff
sc 0 1 2 3 4 sc 0 1 2 3 4 kDa
37
Parp3+/+
Parp3-/-
MyoD
(d) Post diff
(d) Post diff
sc 0 1 2 3 4 sc 0 1 2 3 4 kDa
50
37
Parp3+/+
Parp3-/-
Actin
(d) Post diff
(d) Post diff
sc 0 1 2 3 4 sc 0 1 2 3 4 kDa
50
37
Parp3+/+
Parp3-/-

Fig. S8

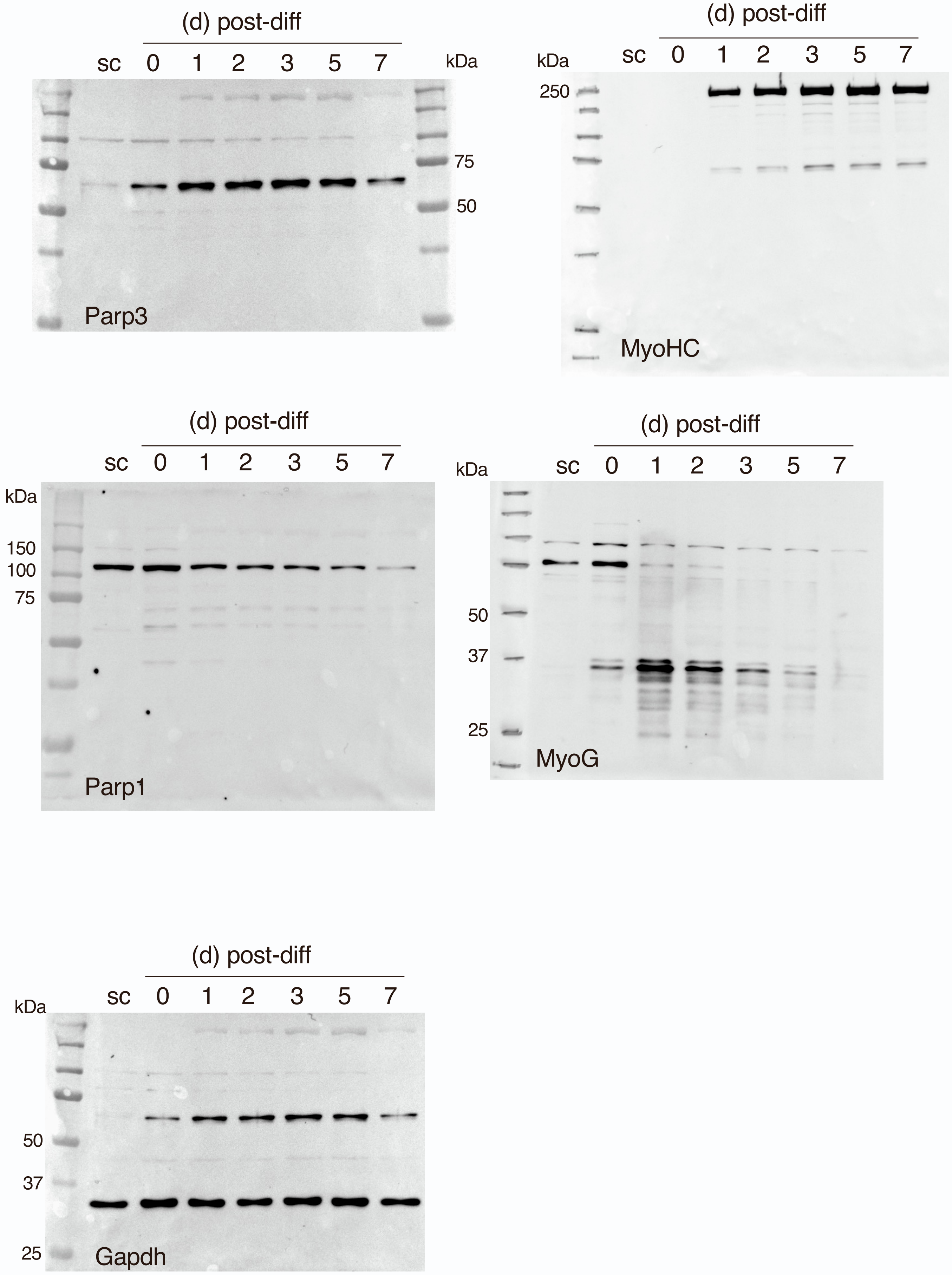

(d) post-diff
sc 0 1 2 3 5 7
kDa
75
50
Parp3
(d) post-diff
sc 0 1 2 3 5 7
kDa
250
MyoHC
(d) post-diff
sc 0 1 2 3 5 7
kDa
150
100
75
Parp1
(d) post-diff
sc 0 1 2 3 5 7
kDa
50
37
25
MyoG
(d) post-diff
sc 0 1 2 3 5 7
kDa
50
37
25
Gapdh

Fig. S9

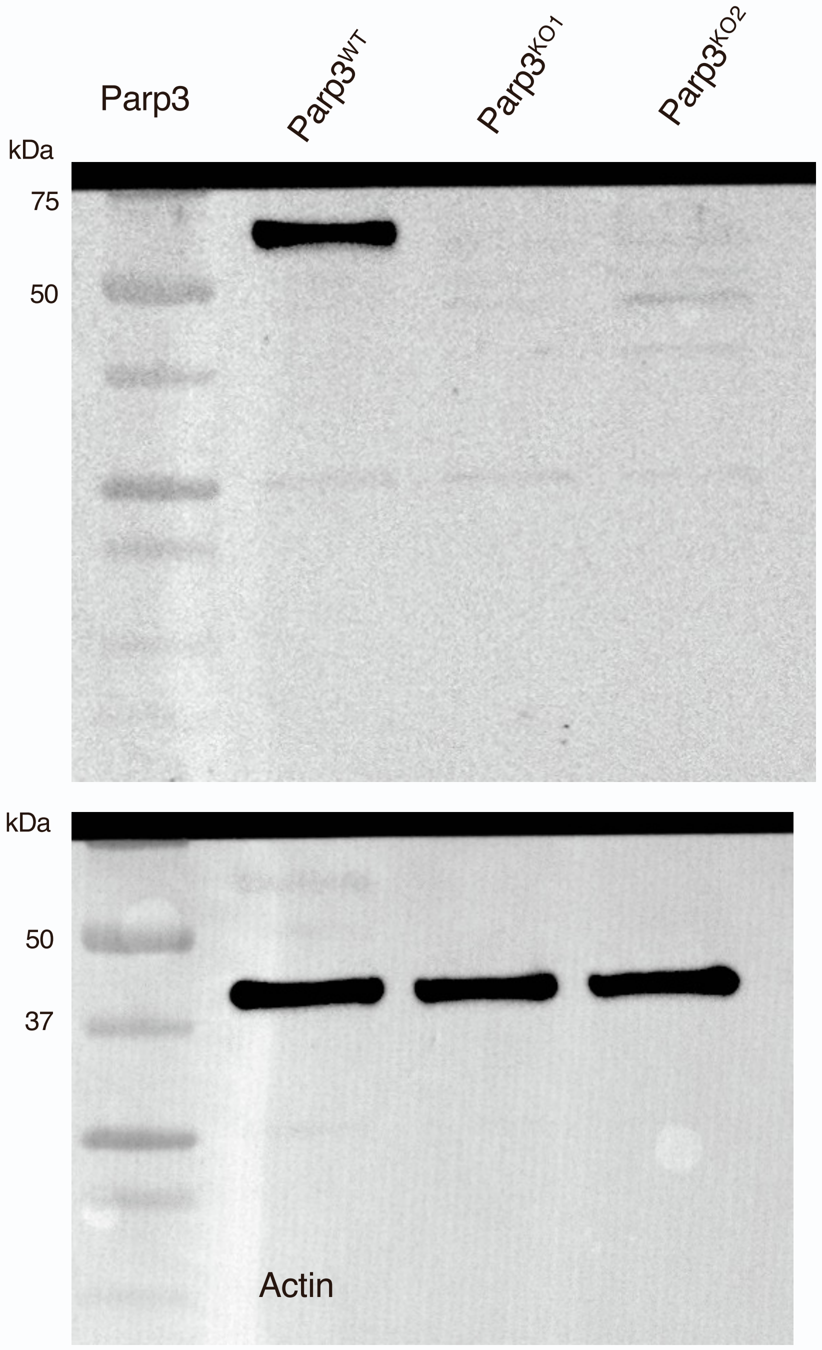

Fig. S10

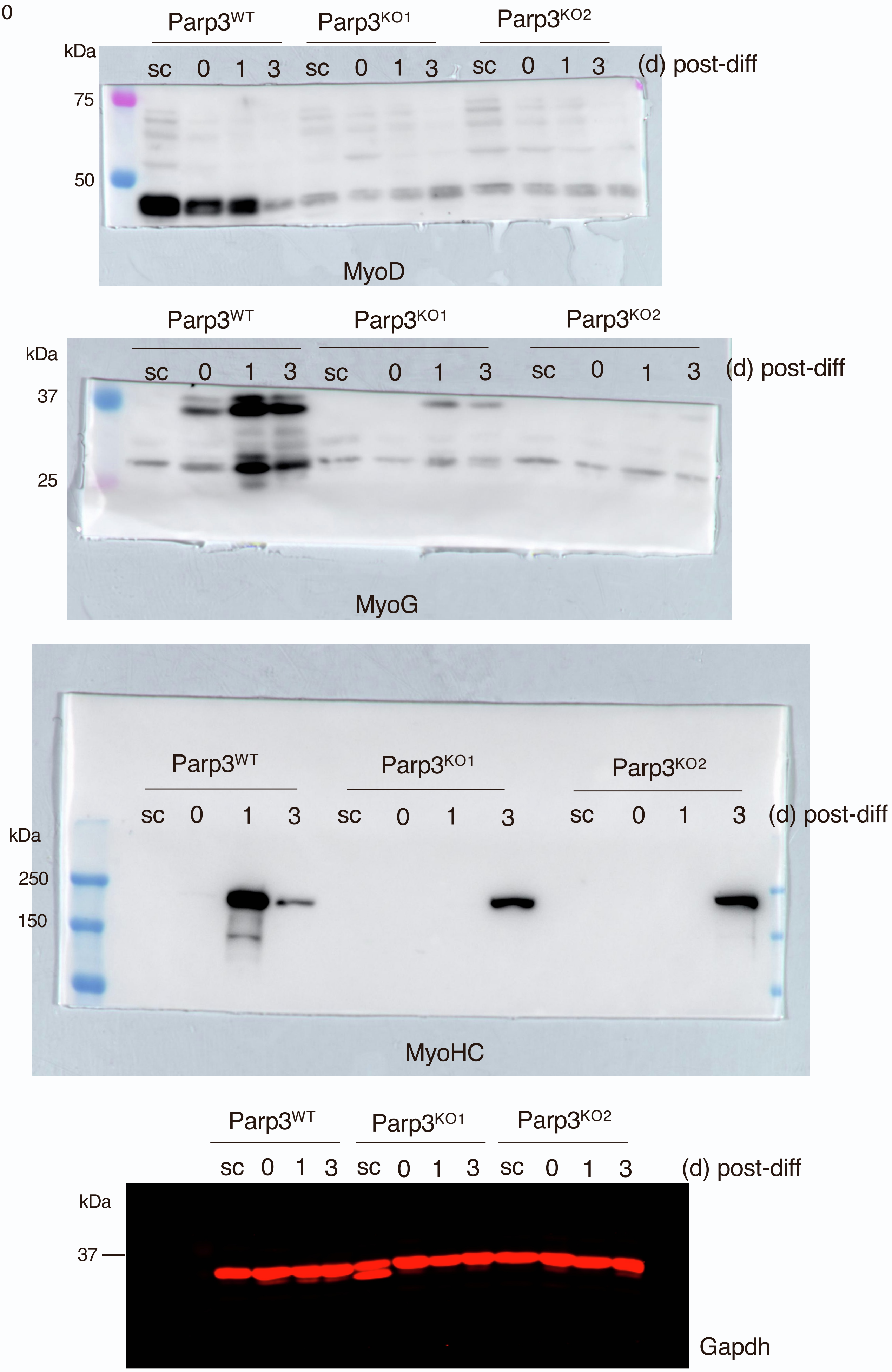

Parp3WT
Parp3KO1
Parp3KO2
kDa
sc 0 1 3 sc 0 1 3 sc 0 1 3 (d) post-diff
75
50
MyoD
Parp3WT
Parp3KO1
Parp3KO2
kDa
sc 0 1 3 sc 0 1 3 sc 0 1 3 (d) post-diff
37
25
MyoG
Parp3WT
Parp3KO1
Parp3KO2
sc 0 1 3 sc 0 1 3 sc 0 1 3 (d) post-diff
kDa
250
150
MyoHC
Parp3WT
Parp3KO1
Parp3KO2
sc 0 1 3 sc 0 1 3 sc 0 1 3 (d) post-diff
kDa
37
Gapdh

Fig. S11

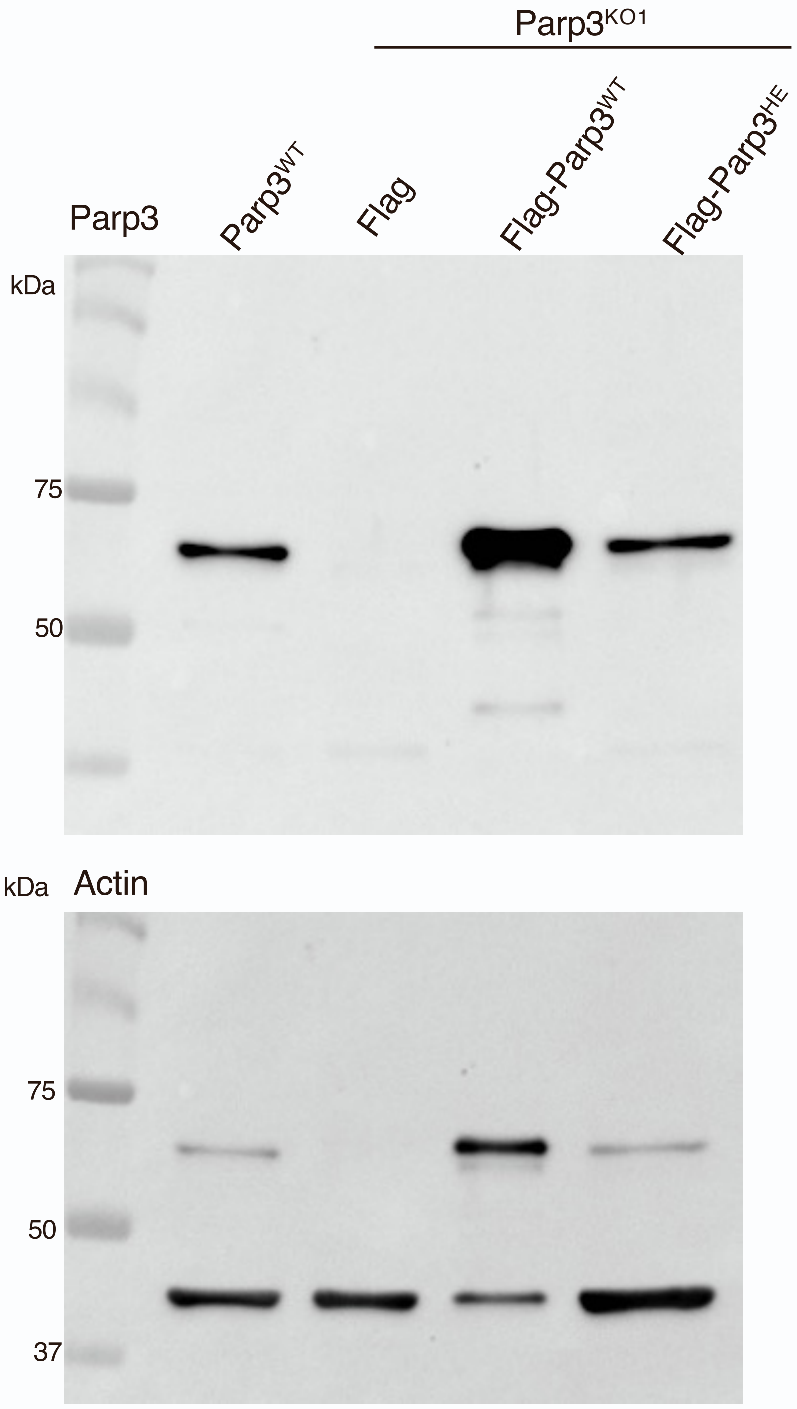

Parp3^KO1
Parp3
Parp3^WT
Flag
Flag-Parp3^WT
Flag-Parp3^HE
kDa
75
50
kDa
Actin
75
50
37

Fig. S12

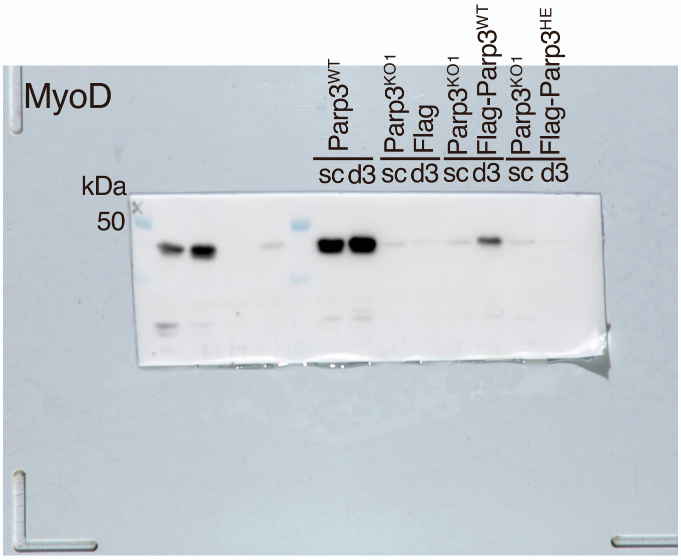
MyoD
kDa
50
Parp3WT
Parp3KO1 Flag
Parp3KO1 Flag-Parp3WT
Parp3KO1 Flag-Parp3HE
sc d3 sc d3 sc d3 sc d3

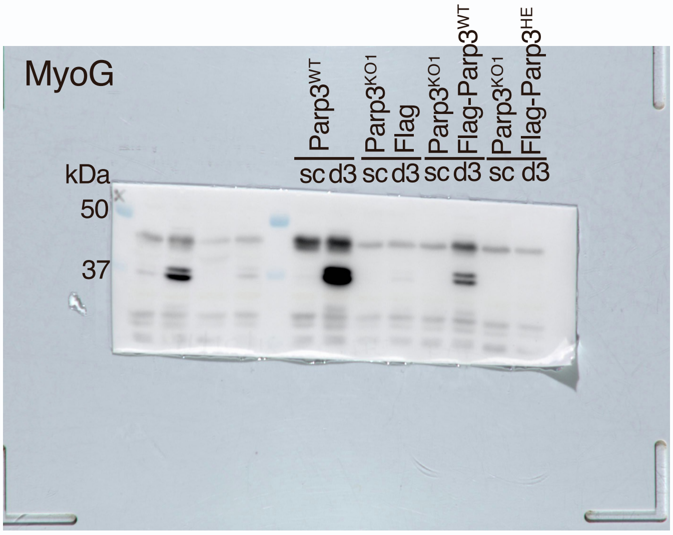
MyoG
kDa
50
37
Parp3WT
Parp3KO1 Flag
Parp3KO1 Flag-Parp3WT
Parp3KO1 Flag-Parp3HE
sc d3 sc d3 sc d3 sc d3

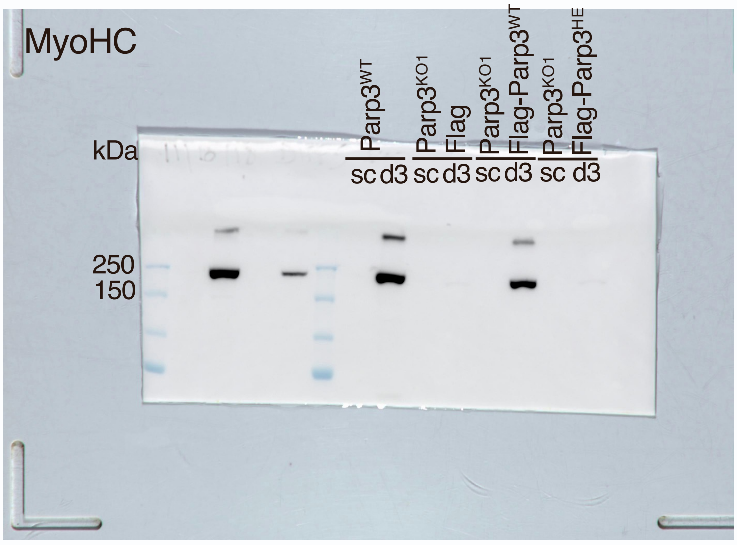
MyoHC
kDa
250
150
Parp3WT
Parp3KO1 Flag
Parp3KO1 Flag-Parp3WT
Parp3KO1 Flag-Parp3HE
sc d3 sc d3 sc d3 sc d3

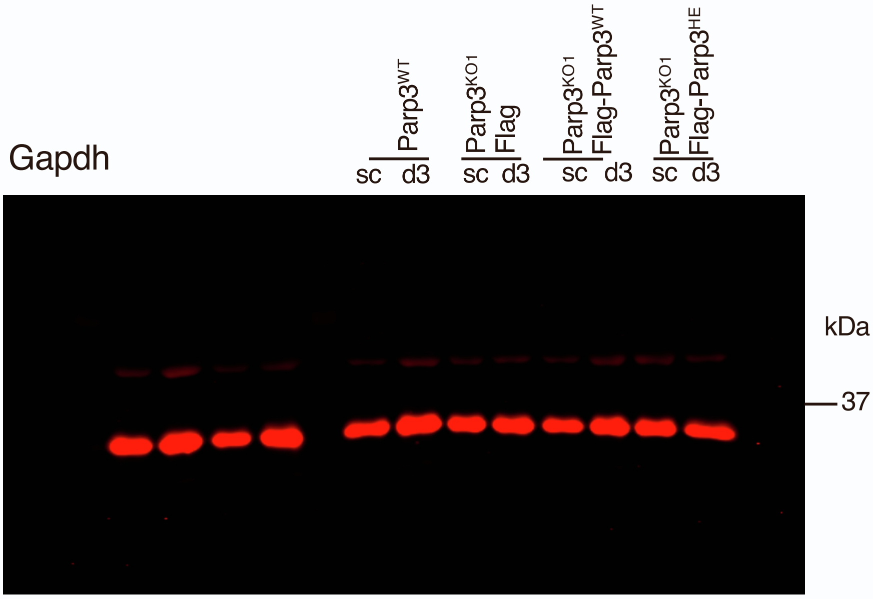
Gapdh
Parp3WT
Parp3KO1 Flag
Parp3KO1 Flag-Parp3WT
Parp3KO1 Flag-Parp3HE
sc d3 sc d3 sc d3 sc d3
kDa
37
